# Solvent-Free Synthesis and Safener Activity of Sulfonylurea Benzothiazolines

**DOI:** 10.3390/molecules22101601

**Published:** 2017-09-22

**Authors:** Ying Fu, Jing-Yi Wang, Dong Zhang, Yu-Feng Chen, Shuang Gao, Li-Xia Zhao, Fei Ye

**Affiliations:** Department of Applied Chemistry, College of Science, Northeast Agricultural University, Harbin 150030, China; fuying@neau.edu.cn (Y.F.); wang-jingyi@foxmail.com (J.-Y.W.); zhangdong-qlc@163.com (D.Z.); cyf1989919@163.com (Y.-F.C.); gaoshuang@neau.edu.cn (S.G.); zhaolixia@neau.edu.cn (L.-X.Z.)

**Keywords:** active subunit combination, sulfonylurea benzothiazoline, solvent-free synthesis, safener activity

## Abstract

A series of novel sulfonylurea benzothiazolines was designed by splicing active groups and bioisosterism. A solvent-free synthetic route was developed for the sulfonylurea benzothiazoline derivatives via the cyclization and carbamylation. All compounds were characterized by IR, ^1^H-NMR, ^13^C-NMR, HRMS. The biological activity tests indicated the compounds could protect maize against the injury caused by chlorsulfuron to some extent. The molecular docking result showed that the new compound competed with chlorsulfuron to bind with the herbicide target enzyme active site to attain detoxification.

## 1. Introduction

Sulfur and nitrogen-containing heterocyclic compounds play a key role in the pharmaceutical and chemical industries [1,2]. In particular, sulfur-containing heterocyclic compounds are widely used in the agricultural field. Several new benzothiazole compounds have been synthesized as potential antimicrobial and antiparasitic agents [3]. 1,2-Benzisothiazolin-3-one, used as a fungicide, shows good sterilization and anti-corrosion performance [4]. Thiazole compounds are also reported to be herbicide safeners [5].

**Scheme 1:**
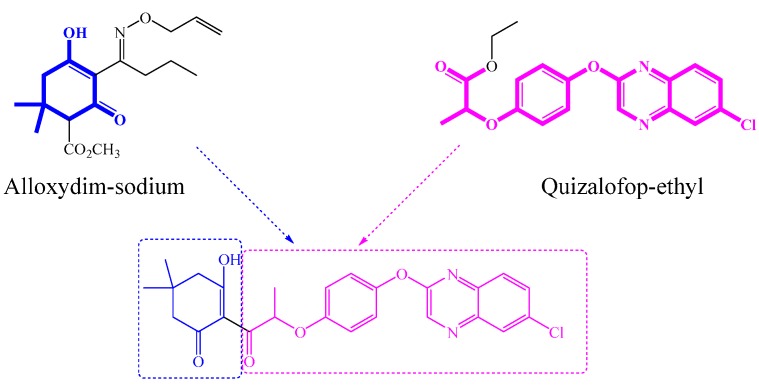
The exploitation of the new herbicide.

Some bioactive compounds have been discovered by combining active subunits of known active molecules. For example, new triketone derivatives with better herbicidal activity have been designed by splicing active group alloxydim-sodium into quizalofop-ethyl (Scheme 1) [6]. Many successful cases have been reported in recent years [7,8].

Herbicides and safeners may share common molecular characteristics, depending on the structure-activity relationships (SAR) and the mechanisms of the safeners [9]. A systematic review of the chemical characteristics and SAR of herbicide safeners indicated that there are closely similar structural features between herbicides and safeners [10]. For example, sulfamide compounds may be an antidote to protect plants from the injury caused by sulfonylurea herbicides.

**Scheme 2:**
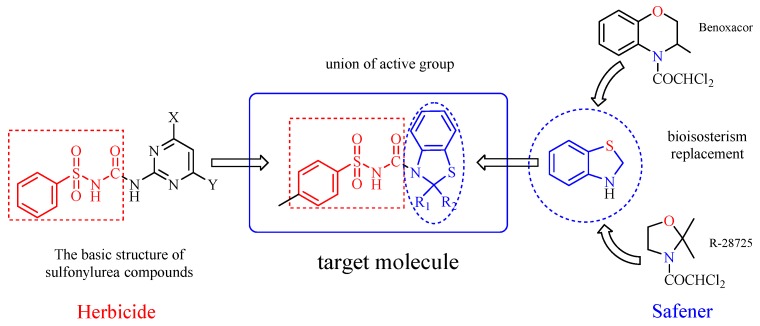
The design of the target compounds.

According to the facts mentioned above, and continuing on from our previous research, a series of sulfonylurea substituted benzothiazoline compounds **3a**–**3m** was designed based on active subunit combination, bioisosteric replacement, and SAR; the sulfur- and nitrogen-containing heterocyclic was retained and modified on the sulfonylurea functional groups (Scheme 2) [11,12,13].

**Scheme 3:**
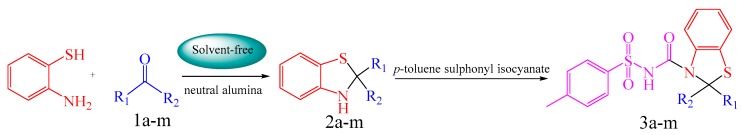
Route for synthesis of the target compounds **3**.

A number of synthetic routes for benzothiazoline have been reported. The most common method is the condensation of *o*-aminothiophenol with carbonyl compounds in the presence of *p*-toluenesulfonic acid with Ga(OTf)_3_ as catalyst [14,15]. Other methods include the reaction of 2,2’-dinitrodiphenyl disulfide with ketones in the presence of TiCl_4_/Sm and SmI_2_/HMPA [16,17]. Nevertheless, all these reported methods suffer from some drawbacks, such as the use of expensive or toxic catalysts, long reaction times, tedious synthetic procedures, or low yields of the products. In recent years, solvent-free organic synthesis has become a popular method, and has attracted immense interest as an environmentally benign method. It leads to good yields, clean reactions, and shorter reaction times [18]. In view of the facts mentioned above, a series of novel sulfonylurea benzothiazoline was designed and synthesized with *o*-aminothiophenol and ketone as starting materials in the presence of neutral alumina via a solvent-free procedure (Scheme 3) [19].

## 2. Results and Discussion

### 2.1. Chemistry

1,3-Benzothiazoline derivatives **2** were synthesized with *o*-aminothiophenol and ketone **1** smoothly in the presence of neutral alumina. All compounds were synthesized under solvent-free conditions and further purified by column chromatography (silica gel, petroleum ether (PE):ethyl acetate; 20:1) to give the pure product.

The yields of compounds **2** were 56–92% (Table 1). The substituent group affected the yields significantly. When the substituents were cyclopentyl or cyclohexyl, the formation of spiro compounds made the structure more stable than others. Thus, the yields of **2l**, **2m** were better, at 91% and 92%, respectively. The bulk substituent reduced the stability of the benzothiazoline. The yield of **2k** was only 56% which might be caused by the R_1_ and R_2_ being two *n*-propyl groups. The yields were similar for **2f** and **2g**.

**Scheme 4:**
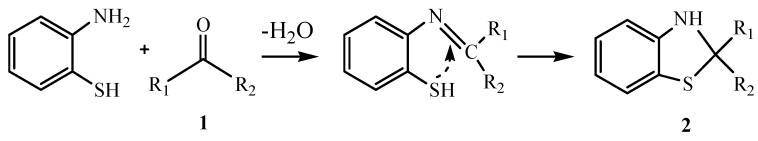
Mechanistic outline for cyclization reaction.

The proposed outline for the cyclization is outlined in Scheme 4. First, *o*-aminothiophenol reacted with ketone to generate a Schiff base. Nucleophilic attack from nearby S atom gave the intermediate **2**.

The target compounds **3** were synthesized by direct carbamylation with 1,3-benzothiazoline derivatives **2** and tosyl isocyanate. All compounds were obtained by washing the mixture with the solution (anhydrous benzene:Anhydrous *n*-hexane; 1:1) to get white powder.

The yields of compounds **3** were 36%–99% (Table 1). When the substituents were symmetric small groups or cyclic structures, the yield was good. Compounds **3a**, **3l**, **3m** were over 90% yields.

In general, there was no significant effect on the yields of the target products caused by steric hindrance, because the substituents at the 2-position were almost perpendicular to the plane of benzothiazoline ring. Comparing compound **3f** and **3g**, it was also noticeable that the presence of a straight-chain at the 2-position of benzothiazoline increased the yield. In contrast, the yields were decreased when there was a branch-chain with the same carbon atom number.

The peaks at 1708, 1341 and 1152 cm^−1^ in the IR for compound **3a** confirmed the presence of the carbonyl and sulfonyl groups. The ^1^H-NMR spectrum also confirmed the proposed structure, the three hydrogens at *δ* 2.37–2.41 ppm showed the methyl of the benzene ring. The signals at *δ* 6.48–7.85 ppm related to the benzene ring. The single signal observed at *δ* 11.86 ppm was characteristic of hydrogen linked to a nitrogen atom.

### 2.2. Biological Activity

All the novel sulfonylurea benzothiazoline derivatives **3** were evaluated for their protection of maize against the injury of chlorsulfuron (2 μg/kg) (Table 2). After a preliminary screening, the concentration of the safener and compounds applied in the bioassay was determined. Chlorsulfuron could provoke an obvious decrease in the growth of maize, but significant differences were observed after the introduction of compounds. The recovery rates of the root length were attained over 25% except for compounds **3e**, **3j**. Recovery rates of plant weight were over 40%, except for compound **3e**. Among all the test compounds, compound **3c** showed the best safener activity against the injury of chlorsulfuron, better than the commercialized safener AD-67. A good protection activity may be due to the introduction of a sulfonyl group, causing the compound to bind with the herbicide target site competitively, and reduce the injury of chlorsulfuron.

**Figure 1:**
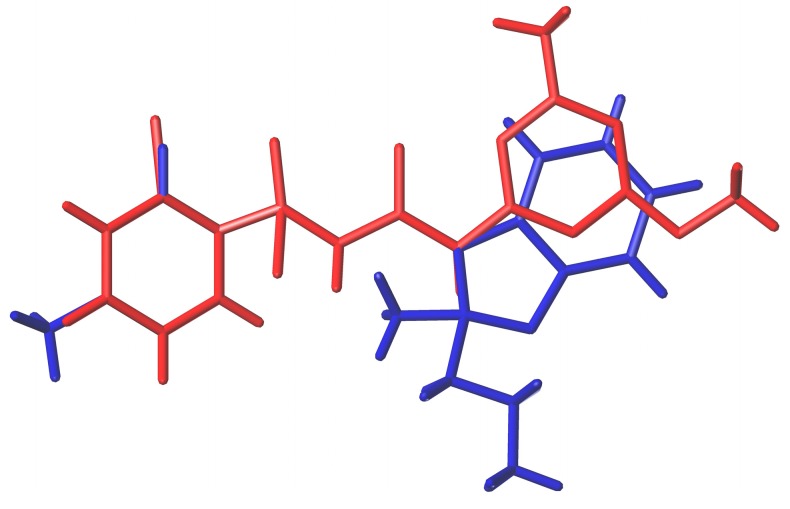
Superimposed molecular structure modeling. The structure of chlorsulfuron is shown in red, and compound **3c** is shown in blue.

Comparing the chemical properties of compound **3c** and chlorsulfuron, such as log *p*, p*K*_a_, molecular weight (MW) and electronegativity, with a view to proving the hypothesis that safeners may act as competitive antagonists for herbicides at the herbicide target site (Table 3), it was observed that p*K*_a_, MW and the electronegativity of compound **3c** were all similar to the herbicide chlorsulfuron. This indicated that, in terms of the investigated features, the safener/herbicide combinations were quite similar at the molecular level. The visual evaluation of the superimposed molecular structures is shown in Figure 1. Chlorsulfuron and **3c** were perfectly aligned in a common skeleton.

**Figure 2:**
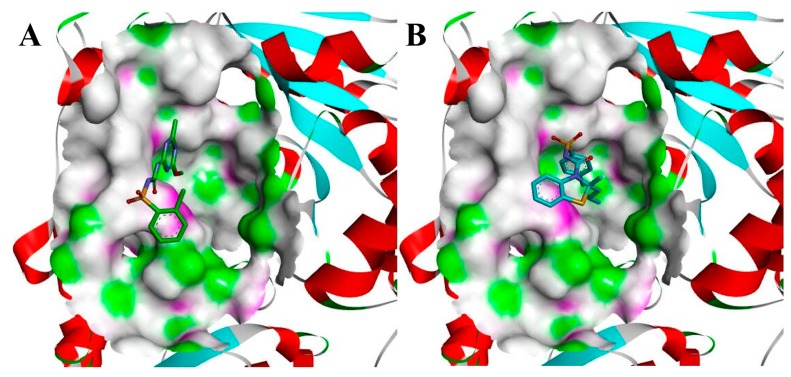
The docking modeling of chlorsulfuron (**A**) and **3c** (**B**) with ALS. The carbon atoms are shown in green (**A**) and light blue (**B**), the sulfur atoms are shown in yellow, the oxygen atoms are shown in red, and the nitrogen atoms are shown in blue.

Sulfonylurea herbicides are a kind of acetolactate synthase (ALS) inhibitors. The three-dimensional structure of compound **3c** and chlorsulfuron was constructed by the sketch module of SYBYL-X 2.0. Subsequently, the molecule was optimized and Gasteiger-Huckel charges were calculated. The crystal structure of ALS was taken from the Protein Data Bank (PDB ID 1YHY). Docking modeling used the CDOCKER method in Accelrys Discovery Studio 2.5 (Accelrys Inc., San Diego, CA, USA, 2005). Before docking, the protein structure was given the CHARMM force field and removed the water and some other co-crystallized small molecules. After the protein preparation, the docking studies active site was defined, with a subset region of 13.0A from the center of the known ligand. The Top Hits was set to 100, and the default values were used for the remaining parameters. The binding energy of the small molecule-receptor protein complex was used as an evaluation index, with the largest negative representation of the most stable conformation. The molecular docking result showed that both compound **3c** and chlorsulfuron were able to bind well to the herbicide target active site of ALS (Figure 2). In the docking modeling, the phenyl moiety of chlorsulfuron rotated to the right side in the active site, effectively blocking the entrance to the channel and preventing the substrate from binding with the active site, caused herbicidal activity. In contrast, the phenyl moiety of compound **3c** turned left at the active site, partially blocking the entrance of the channel. While preventing the combination of chlorsulfuron with the active site, the small substrate had more opportunity to thrust itself into the channel and catalyze the active site.

When compound **3c** was applied before or simultaneously with chlorsulfuron, it potentially competed with chlorsulfuron at the target site by preventing the herbicide from reaching or acting on the ALS active pocket, leading to counter action of the herbicide. This may be the detoxification mechanism of the novel compound.

## 3. Materials and Methods

### 3.1. Reagents and Analysis

All the reagents were analytical grade, and were used without further purification. The IR spectra were recorded on a Bruker ALPHA-T spectrometer (BRUKER Inc., Beijing, China). The ^1^H-NMR and ^13^C-NMR spectra were recorded on a Bruker AVANVE 300 MHz (BRUKER Inc., Beijing, China) using CDCl_3_ (Energy Chemical., Shanghai, China) or DMSO-*d_6_* (Energy Chemical., Shanghai, China) as solvent and TMS (Energy Chemical., Shanghai, China) as internal standard. The melting point was measured on a Beijing Taike melting point apparatus (X-4) (Beijing, China), and was uncorrected. The high-resolution mass spectrometry was recorded on a FT-ICR MS spectrometer (BRUKER Inc., Beijing, China). The spectrogram datas of compounds could be found in the Supplementary Materials.

### 3.2. General Procedure for the Preparation of 1,3-Benzothiazoline Derivatives ***2***

*o*-Aminothiophenol (0.03 mol) and ketone (0.02 mol) were mixed in a round-bottomed flask in the presence of neutral alumina (3 g). The reaction mixture was stirred under nitrogen atmosphere at appropriate temperature for a period of time. The reaction mixture was extracted with chloroform and filtered. The organic layer was dried over anhydrous Na_2_SO_4_ and chloroform was evaporated under vacuum. The pure products were obtained by recrystallization (**2a**, **2e**, **2i**, **2l**–**2m**) or column chromatography on silica gel eluting with PE and EtOAc (20:1) (**2b**–**2d**, **2f**–**2h**, **2j**–**2k**).

*2,2-Dimethylbenzothiazoline* (**2a**): White solid, Yield 82%. IR (KBr, $\bar{\nu }$/cm^−1^) 3334 (N-H), 2961–2917 (C-H), 1581–1363 (C=C). ^1^H-NMR (300 MHz, CDCl_3_): *δ* (ppm) 6.67–7.10 (m, 4H), 3.97 (s, 1H), 1.74 (s, 6H). ^13^C-NMR (75 MHz, CDCl_3_): *δ* (ppm) 145.9, 128.5, 125.1, 122.2, 121.1, 111.5, 74.8, 31.7, 31.7.

*2-Ethyl-2-methyldihydro-benzothiazoline* (**2b**): Yellow liquid, Yield 83%. IR (KBr, $\bar{\nu }$/cm^−1^) 3353 (N-H), 3086–2875 (C-H), 1581–1375 (C=C). ^1^H-NMR (300 MHz, CDCl_3_): *δ* (ppm) 6.60–7.05 (m, 4H), 3.89 (s, 1H), 1.80–2.02 (m, 2H), 1.66 (s, 3H), 1.02–1.06 (t, *J* = 7.5 Hz, 3H). ^13^C-NMR (75 MHz, CDCl_3_): *δ* (ppm) 146.2, 127.7, 125.0, 122.0, 129.5, 110.8, 78.9, 38.9, 29.6, 9.8.

*2-Methyl-2-propylbenzothiazoline* (**2c**): Yellow liquid, Yield 92%. IR (KBr, $\bar{\nu }$/cm^−1^) 3353 (N-H), 3067–2871 (C-H), 1583–1395 (C=C). ^1^H-NMR (300 MHz, CDCl_3_): *δ* (ppm) 6.62–7.06 (m, 4H), 3.85 (s, 1H), 1.80–1.98 (m, 2H), 1.68 (s, 3H), 1.44–1.58 (m, 2H), 0.94–0.99 (t, *J* = 7.5 Hz, 3H). ^13^C-NMR (75 MHz, CDCl_3_): *δ* (ppm) 146.0, 127.9, 124.9, 121.9, 120.6, 110.9, 78.2, 46.4, 30.0, 18.8, 14.2.

*2-Isopropyl-2-methylbenzothiazoline* (**2d**): Yellow liquid, Yield 84%. IR (KBr, $\bar{\nu }$/cm^−1^) 3348 (N-H), 3068–2869 (C-H), 1583–1373 (C=C). ^1^H-NMR (300 MHz, CDCl_3_): *δ* (ppm) 6.61–7.07 (m, 4H), 3.95 (s, 1H), 2.13–2.22 (m, 1H), 1.66 (s, 3H), 1.05–1.11 (q, *J* = 6.6 Hz, 6H). ^13^C-NMR (75 MHz, CDCl_3_): *δ* (ppm) 146.4, 127.0, 124.9, 121.8, 120.1, 110.2, 82.6, 39.9, 27.1, 19.0, 18.2.

*2-(Acetonyl)-2-methylbenzothiazoline* (**2e**): Transparent crystal, Yield 78%. IR (KBr, $\bar{\nu }$/cm^−1^) 3334 (N-H), 3070–2969 (C-H), 1580–1338, 1706 (C=C). ^1^H-NMR (300 MHz, CDCl_3_): *δ* (ppm) 6.52–6.95 (m, 4H), 6.42 (s, 1H), 3.14–3.21 (q, *J* = 17.4 Hz, 2H), 2.12 (S, 3H), 1.619 (s, 3H). ^13^C-NMR (75 MHz, CDCl_3_): *δ* (ppm) 206.1, 145.9, 125.2, 124.9, 121.1, 118.2, 108.8, 74.3, 55.8, 30.8, 29.5.

*2-Butyl-2-methylbenzothiazoline* (**2f**): Yellow liquid, Yield 76%. IR (KBr, $\bar{\nu }$/cm^−1^) 3349 (N-H), 3068–2859 (C-H), 1583–1374 (C=C). ^1^H-NMR (300 MHz, CDCl_3_): *δ* (ppm) 6.64–7.08 (m, 4H), 4.02 (s, 1H), 1.83–1.98 (m, 2H), 1.70 (s, 3H), 1.31–1.54 (m, 4H), 0.92–0.96 (t, *J* = 7.2 Hz, 3H). ^13^C-NMR (75 MHz, CDCl_3_): *δ* (ppm) 145.9, 127.8, 125.0, 122.0, 120.7, 110.9, 78.4, 73.8, 30.0, 27.7, 22.9, 14.1.

*2-Isobutyl-2-methylbenzothiazoline* (**2g**): Yellow liquid, Yield 76%. IR (KBr, $\bar{\nu }$/cm^−1^) 3357 (N-H), 2967 (C-H), 1582–1391 (C=C). ^1^H-NMR (300 MHz, CDCl_3_): *δ* (ppm) 6.64–7.08 (m, 4H), 3.88 (s, 1H), 1.84–1.98 (m, 3H), 1.70 (s, 3H), 1.02–1.04 (d, *J* = 6.3 Hz, 6H). ^13^C-NMR (75 MHz, CDCl_3_): *δ* (ppm) 145.7, 128.1, 125.0, 122.0, 120.6, 111.0, 78.4, 52.1, 30.5, 25.6, 21.5, 21.0.

*2-tert-Butyl-2-methyl benzothiazoline* (**2h**): Yellow liquid,Yield 80%. IR (KBr, $\bar{\nu }$/cm^−1^) 3362 (N-H), 3068–2871 (C-H), 1582–1385 (C=C). ^1^H-NMR (300 MHz, CDCl_3_): *δ* (ppm) 6.59–7.06 (m, 4H), 3.96 (s, 1H), 1.74 (s, 3H), 1.14 (s, 9H). ^13^C-NMR (75 MHz, CDCl_3_): *δ* (ppm) 146.7, 126.5, 124.8, 121.5, 119.7, 109.5, 85.7, 39.9, 27.1, 26.3, 26.3, 26.3.

*2-Methyl-2-phenethylbenzothiazoline* (**2i**): Transparent crystal, Yield 86%. IR (KBr, $\bar{\nu }$/cm^−1^) 3353 (N-H), 3060–2856 (C-H), 1602–1375 (C=C). ^1^H-NMR (300 MHz, CDCl_3_): *δ* (ppm) 6.66–7.38 (m, 9H), 3.90 (s, 1H), 2.89–2.94 (t, *J* = 6.6 Hz, 2H), 2.22–2.30 (m, 2H), 1.82 (s, 3H). ^13^C-NMR (75 MHz, CDCl_3_): *δ* (ppm) 146.1,141.7, 128.5, 128.5, 128.5, 128.5, 127.5, 126.0, 125.1, 122.0, 120.7, 110.8, 76.7, 45.9, 31.9, 30.3.

*2,2-Diethylbenzothiazoline* (**2j**): Yellow liquid, Yield 82%. IR (KBr, $\bar{\nu }$/cm^−1^) 3371 (N-H), 3068–2930 (C-H), 1583–1398 (C=C). ^1^H-NMR (300 MHz, CDCl_3_): *δ* (ppm) 6.59–7.05 (m, 4H), 3.89 (s, 1H), 1.88–1.95 (q, *J* = 7.5 Hz ,4H), 1.01–1.06 (t, *J* = 7.5 Hz, 6H). ^13^C-NMR (75 MHz, CDCl_3_): *δ* (ppm) 146.7, 127.1, 124.8, 121.7, 120.0, 109.9, 82.7, 34.5, 34.5, 9.2, 9.2.

*2,2-Dipropylbenzothiazoline* (**2k**): Yellow liquid, Yield 56%. IR (KBr, $\bar{\nu }$/cm^−1^) 3370 (N-H), 3068–2871 (C-H), 1583–1397 (C=C). ^1^H-NMR (300 MHz, CDCl_3_): *δ* (ppm) 6.59–7.05 (m, 4H), 3.94 (s, 1H), 1.85–1.90 (t, *J* = 7.8 Hz, 3H), 1.41–1.63 (m, 4H), 0.95–1.00 (t, *J* = 7.5 Hz, 6H). ^13^C-NMR (75 MHz, CDCl_3_):*δ* (ppm) 146.5, 127.3, 124.9, 121.7, 120.1, 110.0, 81.5, 44.7, 44.7, 18.2, 18.2, 14.3, 14.3.

*3H-spiro[1,3-benzothiazoline-2,1’-cyclopentane]* (**2l**): Transparent crystal, Yield 91%. IR (KBr, $\bar{\nu }$/cm^−1^) 3354 (N-H), 3066–2871 (C-H), 1581–1320 (C=C). ^1^H-NMR (300 MHz, CDCl_3_): *δ* (ppm) 6.64–7.08 (m, 4H), 4.00 (s, 1H), 2.16–2.25 (m, 2H), 1.99–2.08 (m, 2H), 1.77–1.82 (m, 4H). ^13^C-NMR (75 MHz, CDCl_3_): *δ* (ppm) 145.9, 128.1, 125.0, 121.8, 129.7, 110.9, 84.2, 42.5, 42.5, 22.9, 21.9.

*3H-spiro[1,3-benzothiazoline-2,1’-cyclohexane]* (**2m**): Transparent crystal, Yield 92%. IR (KBr, $\bar{\nu }$/cm^−1^) 3329 (N-H), 3063–2863 (C-H), 1579–1443 (C=C). ^1^H-NMR (300 MHz, CDCl_3_): *δ* (ppm) 6.64–7.06 (m, 4H), 3.94 (s, 1H), 2.20–2.24 (d, *J* = 12.6 Hz, 2H), 1.53–1.81 (m, 7H), 1.23–1.36 (m, 1H). ^13^C-NMR (75 MHz, CDCl_3_): *δ* (ppm) 145.9, 127.1, 125.0, 121.9,120.5, 110.9, 80.0, 40.9, 40.9, 25.0, 24.0, 24.0.

### 3.3. General Procedure for the Preparation of Sulfonylurea Benzothiazoline Derivatives ***3***

The intermediate **2** (5 mmol) was mixed with tosyl isocyanate (5 mmol) in a round-bottomed flask. The mixture was vigorously stirred at 10 °C for 12 h. At the end of the reaction, a solid precipitated. Then, the solid was washed with the mixed solution of anhydrous benzene and *n*-hexane to get compounds **3**.

*2,2-Dimethyl-N-[(4-methylphenyl)sulfonyl]-1,3-benzothiazoline-3(2H)-formamide* (**3a**): White solid, Yield 96%, m.p. 129–131 °C. IR (KBr, $\bar{\nu }$/cm^−1^) 3242 (N-H), 3071–2928 (C-H), 1708 (C=O), 1341, 1152 (O=S=O). ^1^H-NMR (300 MHz, DMSO-*d*_6_): *δ* (ppm) 11.86 (s, 1H), 6.48–7.85 (m, 8H), 2.37–2.41 (d, *J* = 10.5 Hz, 3H), 1.61–1.70 (d, *J* = 24.9 Hz ,6H). ^13^C-NMR (75 MHz, DMSO-*d*_6_): *δ* (ppm) 147.3, 142.3, 141.9, 129.7, 127.8, 126.1, 125.4, 124.3, 123.5, 121.9, 118.8, 116.4, 109.4, 75.3, 31.9, 23.0, 21.4. HR-MS (ESI): *m*/*z* calcd. for C_17_H_1__9_N_2_O_3_S_2_ ([M + H]^+^) 363.0832 found 363.0827.

*2-Ethyl-2-methyl-N-[(4-methylphenyl)sulfonyl]-1,3-benzothiazoline-3(2H)-formamide* (**3b**): White solid, Yield 65%, m.p. 91–93 °C. IR (KBr, $\bar{\nu }$/cm^−1^) 3234 (N-H), 3064–2934 (C-H), 1704 (C=O), 1350, 1155 (O=S=O). ^1^H-NMR (300 MHz, CDCl_3_): *δ* (ppm) 6.97–7.98 (m, 8H), 2.43–2.45 (d, *J* = 6.3 Hz, 3H), 2.18–2.28 (m, 1H), 1.86–1.98 (m, 1H), 1.77 (s, 3H), 0.78–0.83 (t, *J* = 7.2 Hz, 3H). ^13^C-NMR (75 MHz, CDCl_3_): *δ* (ppm) 146.9, 122.9, 138.1, 136.0, 129.6, 129.6, 128.4, 128.4, 126.5, 125.6, 124.8, 123.5, 115.4, 82.8, 32.6, 26.7, 21.7, 9.3. HR-MS (ESI): *m*/*z* calcd. for C_18_H_2__1_N_2_O_3_S_2_ ([M + H]^+^) 377.0988 found 377.0984.

*2-Propyl-2-methyl-N-[(4-methylphenyl)sulfonyl]-1,3-benzothiazoline-3(2H)-formamide* (**3c**): White solid, Yield 78%, m.p. 91–94 °C. IR (KBr, $\bar{\nu }$/cm^−1^) 3380 (N-H), 3057–2872 (C-H), 1715 (C=O), 1349, 1162 (O=S=O). ^1^H-NMR (300 MHz, CDCl_3_): *δ* (ppm) 6.97–7.98 (m, 8H), 2.44–2.45 (d, *J* = 4.5 Hz, 3H), 2.13–2.23 (m, 1H), 1.82–1.88 (m, 1H), 1.77 (s, 3H), 1.34–1.46 (m, 1H), 0.93–1.03 (m, 1H), 0.70–0.74 (t, *J* = 6.6 Hz, 3H). ^13^C-NMR (75 MHz, CDCl_3_): *δ* (ppm) 146.9, 144.9, 138.0, 135.9, 129.5, 129.5, 128.4, 128.4, 126.5, 125.6, 124.8, 123.5, 115.4, 81.9, 41.6, 27.3, 21.7, 18.4, 13.7. HR-MS (ESI): *m*/*z* calcd. for C_19_H_23_N_2_O_3_S_2_ ([M + H]^+^) 391.1145 found 391.1144.

*2-Isopropyl-2-methyl-N-[(4-methylphenyl)sulfonyl]-1,3-benzothiazoline-3(2H)-formamide* (**3d**): White solid, Yield 70%, m.p. 105–109 °C. IR (KBr, $\bar{\nu }$/cm^−1^) 3347 (N-H), 3056–2973 (C-H), 1706 (C=O), 1357, 1164 (O=S=O). ^1^H-NMR (300 MHz, CDCl_3_): *δ* (ppm) 6.96–7.97 (m, 8H), 2.60–2.69 (m, 1H), 2.45 (s, 3H), 1.79 (s, 3H), 0.92–0.95 (d, *J* = 6.9 Hz, 3H), 0.76–0.78 (d, *J* = 6.6 Hz, 3H). ^13^C-NMR (75 MHz, CDCl_3_): *δ* (ppm) 147.2, 144.8, 138.9, 136.1, 129.5, 129.5, 128.4, 128.4, 126.5, 125.5, 124.7, 123.1, 115.3, 87.1, 36.4, 25.4, 21.7, 18.4, 17.9. HR-MS (ESI): *m*/*z* calcd. for C_19_H_23_N_2_O_3_S_2_ ([M + H]^+^) 391.1145 found 391.1149.

*2-Acetonyl-2-methyl-N-[(4-methylphenyl)sulfonyl]-1,3-benzothiazoline-3(2H)-formamide* (**3e**): White solid, Yield 55%, m.p. 128–130 °C. IR (KBr, $\bar{\nu }$/cm^−1^) 3175 (N-H), 1714 (C=O), 1350, 1164 (O=S=O). ^1^H-NMR (300 MHz, CDCl_3_): *δ* (ppm) 6.52–7.71 (m, 8H), 6.43 (s, 1H), 3.14–3.21 (q, *J* = 17.4 Hz, 4H), 2.37 (s, 3H), 2.12 (s, 3H), 1.61 (s, 3H). ^13^C-NMR (75 MHz, CDCl_3_): *δ* (ppm) 206.1, 145.9, 141.8, 141.4, 129.2, 129.2, 128.3, 125.6, 125.6, 125.2, 124.9, 121.1, 118.2, 108.8, 74.3, 55.8, 30.8, 29.5, 20.8. HR-MS (ESI): *m*/*z* calcd.. for C_19_H_20_N_2_NaO_4_S_2_ ([M + Na]^+)^ 427.0756 found 427.0754.

*2-Butyl-2-methyl-N-butyl-[(4-methylphenyl)sulfonyl]-1,3-benzothiazoline-3-(2H)-formamide* (**3f**): White solid, Yield 50%, IR (KBr, $\bar{\nu }$/cm^−1^) 3245 (N-H), 3068–2871 (C-H), 1702 (C=O), 1352, 1166 (O=S=O). ^1^H-NMR (300 MHz, CDCl_3_): *δ* (ppm) 6.97–7.97 (m, 8H), 2.45 (s, 3H), 1.77 (s, 3H), 0.87–1.42 (m, 6H), 0.69–0.74 (t, *J* = 7.2 Hz, 3H). ^13^C-NMR (75 MHz, CDCl_3_): *δ* (ppm) 144.8, 129.5, 129.5, 128.3, 128.3, 126.4, 125.5, 124.7, 123.4, 121.9, 120.5, 115.4, 110.8, 82.1, 43.8, 38.9, 30.0, 27.2, 22.4, 13.8. HR-MS (ESI): *m*/*z* calcd. for C_20_H_25_N_2_O_3_S_2_ ([M + H]^+^) 405.1301 found 405.1306.

*2-Isobutyl-2-methyl-N-[(4-methylphenyl)sulfonyl]-1,3-benzothiazoline-3-(2H)-formamide* (**3g**): White solid, Yield 36%, m.p. 96–98 °C. IR (KBr, $\bar{\nu }$/cm^−1^) 3248 (N-H), 2976–2864 (C-H), 1697 (C=O), 1345, 1163 (O=S=O). ^1^H-NMR (300 MHz, CDCl_3_): *δ* (ppm) 6.98–7.98 (m, 8H), 2.45 (s, 3H), 2.07–2.13 (q, *J* = 5.1 Hz, 1H), 1.83–1.89 (q, *J* = 5.7 Hz, 1H), 1.80 (s, 3H), 1.64–1.72 (m, 1H), 0.87–0.90 (d, *J* = 6.6 Hz, 3H), 0.70–0.72 (d, *J* = 6.9 Hz, 3H). ^13^C-NMR (75 MHz, CDCl_3_): *δ* (ppm) 147.0, 144.9, 138.0, 136.1, 129.5, 129.5, 128.4, 128.4, 126.5, 125.6, 124.9, 123.5, 115.6, 82.0, 47.7, 27.3, 25.3, 24.3, 23.9, 21.7. HR-MS (ESI): *m*/*z* calcd. for C_20_H_25_N_2_O_3_S_2_ ([M + H]^+^) 405.1301 found 405.1307.

*2-Tert-butyl-2-methyl-N-[(4-methylphenyl)sulfonyl]-1,3benzothiazoline-3(2H)-formamide* (**3h**): White solid, Yield 59%, m.p. 126–129 °C. IR (KBr, $\bar{\nu }$/cm^−1^) 3256 (N-H), 2972 (C-H), 1701 (C=O), 1349, 1167 (O=S=O). ^1^H-NMR (300 MHz, CDCl_3_): *δ* (ppm) 7.03–7.97 (m, 8H), 2.45 (s, 3H), 1.79 (s, 3H), 0.96 (s, 9H). ^13^C-NMR (75 MHz, CDCl_3_): *δ* (ppm) 147.9, 144.7, 140.9, 136.2, 133.1, 129.5, 129.5, 128.3, 128.3, 126.5, 125.6, 122.1, 117.3, 89.4, 43.7, 25.6, 25.6, 25.6, 23.0, 21.7. HR-MS (ESI): *m*/*z* calcd. for C_20_H_25_N_2_O_3_S_2_ ([M + H]^+^) 405.1301 found 405.1305.

*2-Phenylethyl-2-methyl-N-[(4-methylphenyl)sulfonyl]-1,3–benzothiazoline-3(2H)-formamide* (**3i**): White solid, Yield 95%, m.p. 126–129 °C. IR (KBr, $\bar{\nu }$/cm^−1^) 3333 (N-H), 3070–2892 (C-H), 1706 (C=O), 1364, 1118 (O=S=O). ^1^H-NMR (300 MHz, DMSO-*d*_6_): *δ* (ppm) 12.03 (s, 1H), 6.72–7.92 (m, 13H), 2.70–2.82 (m, 1H), 2.48 (s, 1H), 2.42 (s, 3H), 1.99–2.14 (m, 2H), 1.68–1.72 (s, 3H). ^13^C-NMR (75 MHz, DMSO-*d*_6_): *δ* (ppm) 149.0, 147.6, 144.2, 142.2, 141.3, 138.9, 137.8, 129.9, 128.5, 128.1, 127.2, 126.2, 125.5, 124.2, 123.5, 121.7, 118.5, 116.3, 108.9, 80.9, 46.1, 31.7, 29.0, 21.6. HR-MS (ESI): *m*/*z* calcd. for C_24_H_24_N_2_NaO_3_S_2_ ([M + Na]^+^) 475.1120 found 475.1113.

*2,2,-Diethyl-N-[(4-methylphenyl)sulfonyl]-1,3-benzothiazoline-3(2H)-formamide* (**3j**): White solid, Yield 40%, m.p. 103–105 °C. IR (KBr, $\bar{\nu }$/cm^−1^) 3280 (N-H), 2959–2934 (C-H), 1715 (C=O), 1349, 1163 (O=S=O). ^1^H-NMR (300 MHz, CDCl_3_): *δ* (ppm) 6.98–7.98 (m, 8H), 2.46 (s, 3H), 2.13–2.24 (m, 1H), 1.83–1.88 (m, 1H), 1.77 (s, 3H), 1.34–1.46 (m, 1H), 0.93–1.03 (m, 1H), 0.70–0.74 (t, *J* = 7.2 Hz, 3H). ^13^C-NMR (75 MHz, CDCl_3_): *δ* (ppm) 146.9, 144.9, 138.0, 135.9, 129.5, 129.5, 128.4, 128.4, 126.5, 125.6, 124.8, 123.5, 115.3, 81.9, 41.6, 27.3, 21.7, 18.4, 13.7. HR-MS (APCI): *m*/*z* calcd. for C_19_H_23_N_2_O_3_S_2_ ([M + H]^+^) 391.1145 found 391.1140.

*2,2,-Dipropyl-N-[(4-methylphenyl)sulfonyl]-1,3-benzothiazoline-3(2H)-formamide* (**3k**): White solid, Yield 82%, m.p. 101–104 °C. IR (KBr, $\bar{\nu }$/cm^−1^) 3187 (N-H), 3066–2871 (C-H), 1700 (C=O), 1344, 1167 (O=S=O). ^1^H-NMR (300 MHz, CDCl_3_): *δ* (ppm) 6.94–7.96 (m, 8H), 2.44 (s, 3H), 2.15–2.25 (m, 2H), 1.67–1.77 (m, 2H), 1.35–1.47 (m, 2H), 0.81–0.98 (m, 2H), 0.66–0.71 (t, *J* = 7.2 Hz, 6H). ^13^C-NMR (75 MHz, CDCl_3_): *δ* (ppm) 147.0, 144.9, 138.5, 135.7, 129.4, 129.4, 128.6, 128.6, 126.5, 125.4, 124.6, 123.1, 114.8, 85.7, 41.4, 41.4, 21.6, 17.9, 17.9, 13.7, 13.7. HR-MS (ESI): *m*/*z* calcd. for C_21_H_27_N_2_O_3_S_2_ ([M + H]^+^) 419.1458 found 419.1461.

*N-[(4-Methylphenyl)sulfonyl]-3h-screw[1,3-benzothiazoline-2,1’-cyclopentane]-3-formamide* (**3l**): White solid, Yield 95%, m.p. 120–121 °C. IR (KBr, $\bar{\nu }$/cm^−1^) 3286 (N-H), 3066–2869 (C-H), 1602 (C=O), 1343, 1153 (O=S=O). ^1^H-NMR (300 MHz, DMSO-*d*_6_): *δ* (ppm) 6.99–7.99 (m, 8H), 2.53–2.63 (m, 2H), 2.46 (s, 3H), 1.65–2.02 (m, 6H). ^13^C-NMR (75 MHz, DMSO-*d*_6_): *δ* (ppm) 146.8, 144.9, 138.5, 136.1, 129.6, 129.6, 128.4, 128.4, 126.5, 125.7, 124.9, 123.5, 115.5, 87.2, 38.1, 38.1, 23.7, 23.7, 21.7. HR-MS (ESI): *m*/*z* calcd. for C_19_H_2__1_N_2_O_3_S_2_ ([M + H]^+^) 389.0988 found 389.0982.

*N-[(4-Methyl-phenyl)sulfonyl]-3h-screw[1,3-benzothiazoline-2,1’-cyclohexane]-3-formamide* (**3m**): White solid, Yield 99%, m.p. 149–152 °C. IR (KBr, $\bar{\nu }$/cm^−1^) 3287 (N-H), 3071–2849 (C-H), 1692 (C=O), 1346, 1160 (O=S=O). ^1^H-NMR (300 MHz, DMSO-*d*_6_): *δ* (ppm) 11.95 (s, 1H), 6.96–7.89 (m, 8H), 2.44 (s, 3H), 1.09–2.38 (m, 10H). ^13^C-NMR (75 MHz, DMSO-*d*_6_): *δ* (ppm) 149.0, 144.2, 140.0, 137.9, 129.9, 127.8, 126.9, 124.1, 123.3, 121.7, 118.4, 116.4, 109.1, 84.9, 35.1, 25.0, 24.7, 24.3, 24.0, 21.6. HR-MS (ESI): *m*/*z* calcd. for C_20_H_2__3_N_2_O_3_S_2_ ([M + H]^+^) 403.1145 found 403.1149.

## 4. Conclusions

In conclusion, a series of novel sulfonylurea benzothiazoline derivatives was rationally designed and synthesized using a solvent-free method, and identified as potential herbicide safeners for sulfonylurea herbicides. All the synthesized compounds displayed safener activity to chlorsulfuron to some extent, and compound **3c** was even superior to the commercial safener AD-67. The results suggest that compound **3c** might be a novel candidate for a potential safener.

## Figures and Tables

**Table 1:** Characterization data, reaction condition and yields for products **2** and **3**.

Entry	R_1_	R_2_	Compound 2			Compound 3		
			*T* (°C)	Time (h)	Yield (%) ^#^	*T* (°C)	Time (h)	Yield (%) ^#^
a	CH_3_	CH_3_	r.t.	0.5	82	12	12	96
b	CH_3_	CH_2_CH_3_	r.t.	0.5	83	r.t.	5	65
c	CH_3_	CH_2_CH_2_CH_3_	r.t.	0.5	92	r.t.	5	78
d	CH_3_	CH(CH_3_)_2_	80	1	84	r.t.	12	70
e	CH_3_	CH_2_COCH_3_	50	3	78	r.t.	10	55
f	CH_3_	CH_2_CH_2_CH_2_CH_3_	r.t.	2	76	r.t.	10	50
g	CH_3_	CH_2_CH(CH_3_)_2_	r.t.	0.5	76	r.t.	12	36
h	CH_3_	C(CH_3_)_3_	88	1	80	36	2	59
i	CH_3_	PhCH_2_CH_2_	r.t.	0.5	86	12	12	95
j	CH_2_CH_3_	CH_2_CH_3_	r.t.	0.5	82	5	5	40
k	CH_2_CH_2_CH_3_	CH_2_CH_2_CH_3_	80	0.5	56	4	4	82
l	(CH_2_)_4_		r.t.	0.5	91	12	12	95
m	(CH_2_)_5_		r.t.	0.5	92	12	12	99

^#^ Refers to yields of crude products only; r.t.: Room temprature.

**Table 2:** Effect of detoxification of compounds **3a**–**m** to growth index of maize ^i, ii, iii, iv^.

Compound	Root Length Recovery Rate (%)	Plant Height Recovery Rate (%)	Root Fresh Weight Recovery Rate (%)	Plant Fresh Weight Recovery Rate (%)
**AD-67**	32.66 ^efg^	81.62 ^de^	81.25 ^b^	89.47 ^c^
**3a**	38.05 ^cde^	68.57 ^f^	37.50 ^h^	57.89 ^ef^
**3b**	35.02 ^def^	54.41 ^g^	43.75 ^g^	21.05 ^h^
**3c**	55.89 ^a^	109.56 ^a^	87.50 ^b^	126.31 ^a^
**3d**	42.76 ^bc^	100.92 ^ab^	6.25 ^m^	52.63 ^f^
**3e**	19.87 ^i^	20.59 ^i^	56.25 ^e^	42.11 ^g^
**3f**	56.23 ^a^	91.73b ^cd^	12.50 ^l^	73.68 ^d^
**3g**	27.95 ^gh^	81.86 ^de^	37.50 ^h^	84.21 ^c^
**3h**	45.12 ^b^	72.24 ^ef^	87.50 ^b^	105.26 ^b^
**3i**	32.66 ^efg^	84.01 ^cd^	31.25 ^i^	110.53 ^b^
**3j**	18.18 ^ij^	73.35 ^ef^	156.25 ^a^	57.89 ^ef^
**3k**	31.31 ^fg^	40.26 ^h^	37.50 ^h^	57.89 ^ef^
**3l**	33.00 ^efg^	89.34 ^cd^	18.75 ^k^	84.21 ^c^
**3m**	25.93 ^h^	92.65 ^bc^	12.50 ^l^	110.53 ^b^

^i^ Data are means of three replicates; ^ii^ $\mathrm{Recovery}\;\mathrm{Rate}\;(\%)\;=\;\frac{\mathrm{Treated}\;\mathrm{with}\;\mathrm{compounds}\;-\;\mathrm{Treated}\;\mathrm{with}\;\mathrm{chlorsulfuron}\;}{\;\mathrm{Contrast}\;-\;\mathrm{Treated}\;\mathrm{with}\;\mathrm{chlorsulfuron}}$; ^iii^ Water treated was used as contrast; ^iv^ Small letter is significant at the 0.05 level.

**Table 3:** Chemical property comparisons of chlorsulfuron with **3c**.

Compounds	log *p* ^a^	p*K*_a_^b^	MW	Electronegativity ^c^
Chlorsulfuron	2.63	4.1 ± 0.4	357.77	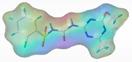
**3c**	4.85	4.6 ± 0.4	390.52	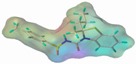

^a^ The log *p* was predicted by ChemBioOffice 2014; ^b^ The p*K*_a_was predicted by ACD/lab; ^c^ The Electronegativity was predicted by Sybyl-X 2.0 (Tripos Inc., St. Louis, MO, USA).

## Supplementary Materials

Supplementary data associated with this article can be found in the online.
