# Supplementary material for: Solvent-Free Synthesis and Safener Activity of Sulfonylurea Benzothiazolines
Source: Molecules. 2017 Sep 22;22(10):1601. doi: 10.3390/molecules22101601 (PMC6151413; doi:10.3390/molecules22101601)
Supplement: Supplementary file 1 [file molecules-22-01601-s001.pdf]

2,2-dimethyl-N-[(4-methylphenyl)sulfonyl]-1,3-benzothiazoline-3(2H)-formamide (**3a**)

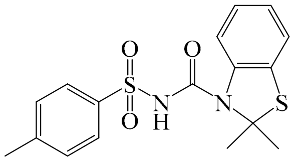

IR:

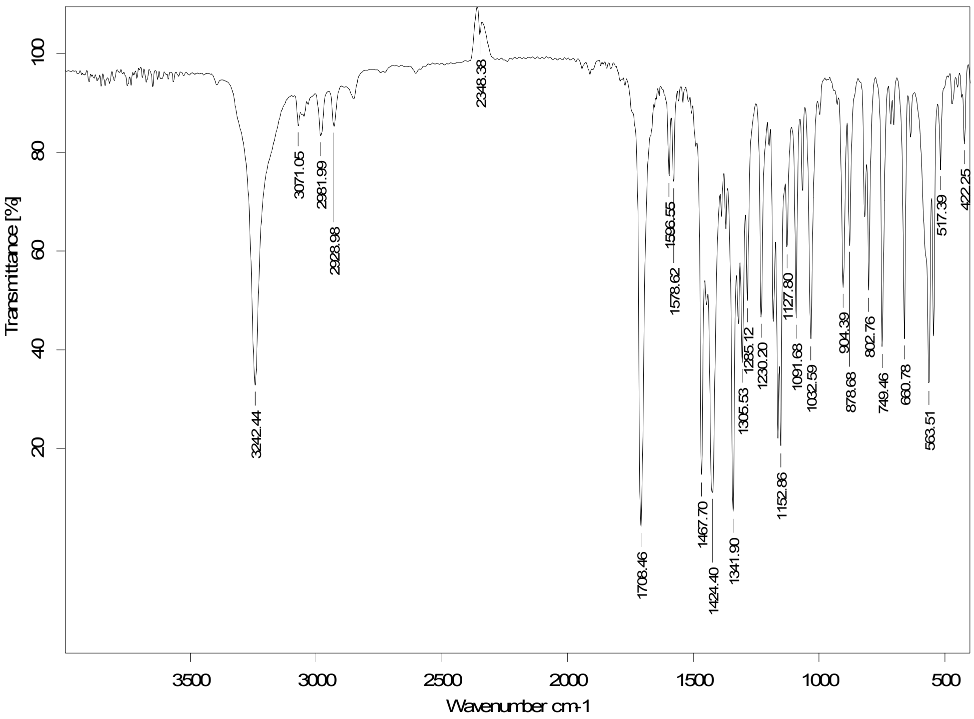

^{1}H-NHR:

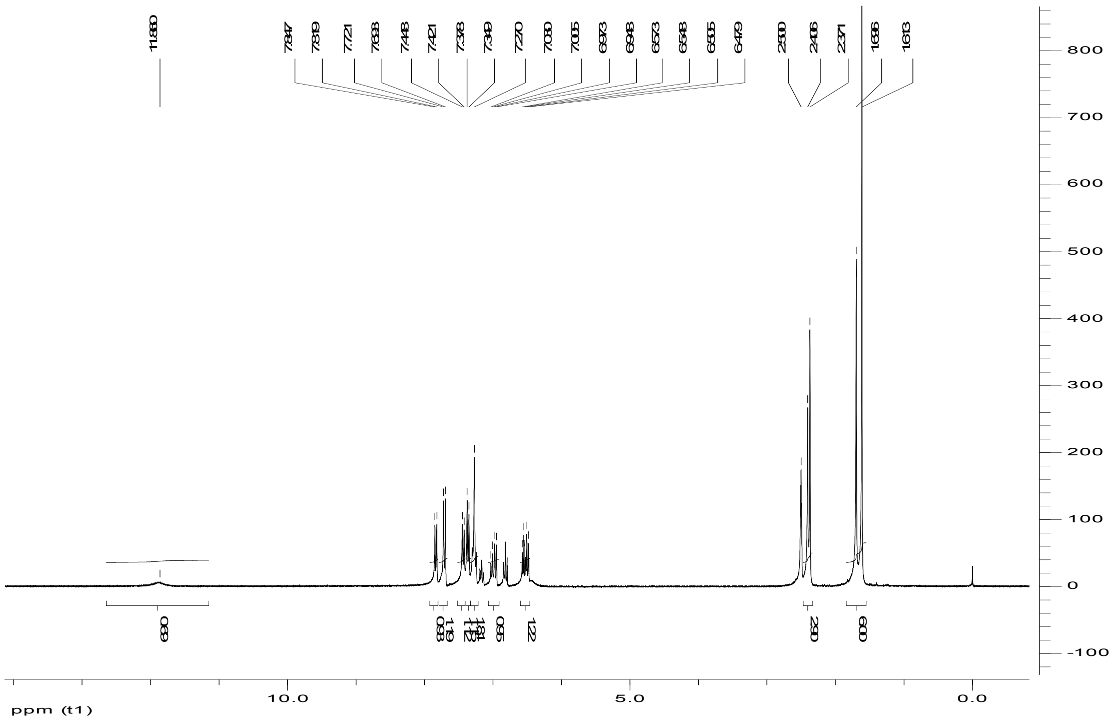

^{13}C-NHR:

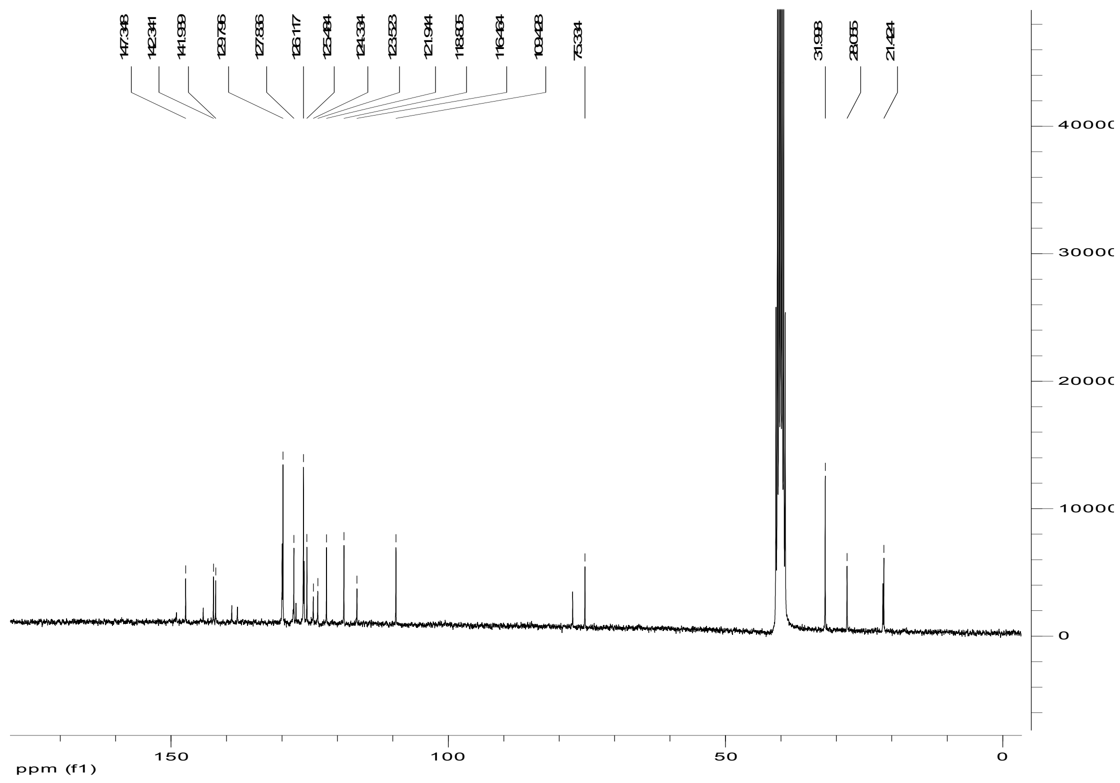

The HRMS was not obtained.

2-ethyl-2-methyl-N-[(4-methylphenyl)sulfonyl]-1,3-benzothiazoline-3(2H)-form amide (**3b**)

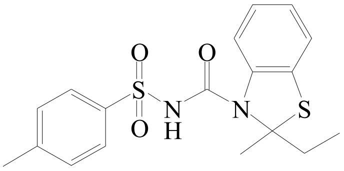

IR:

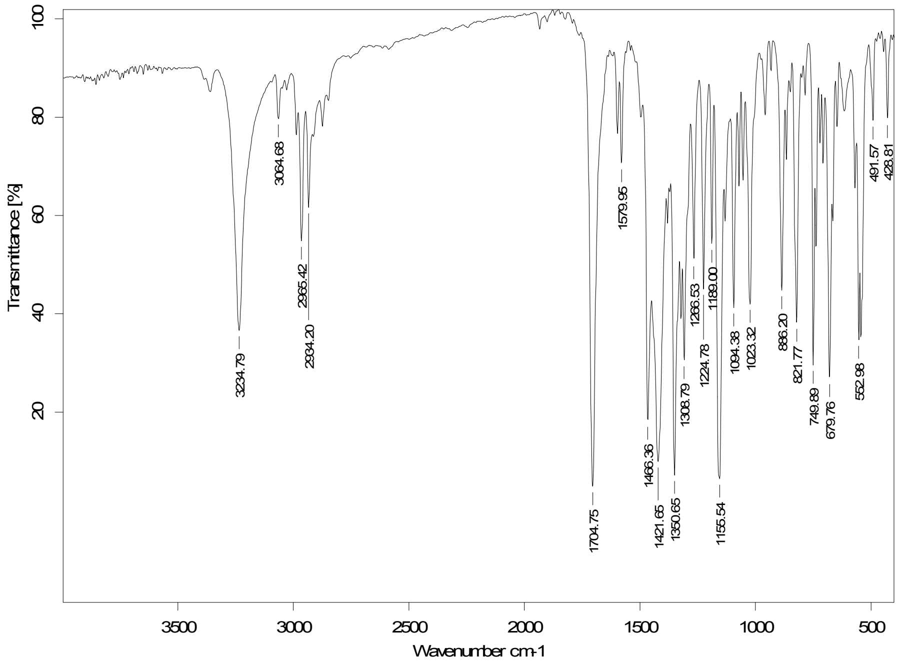

[1]H-NHR:

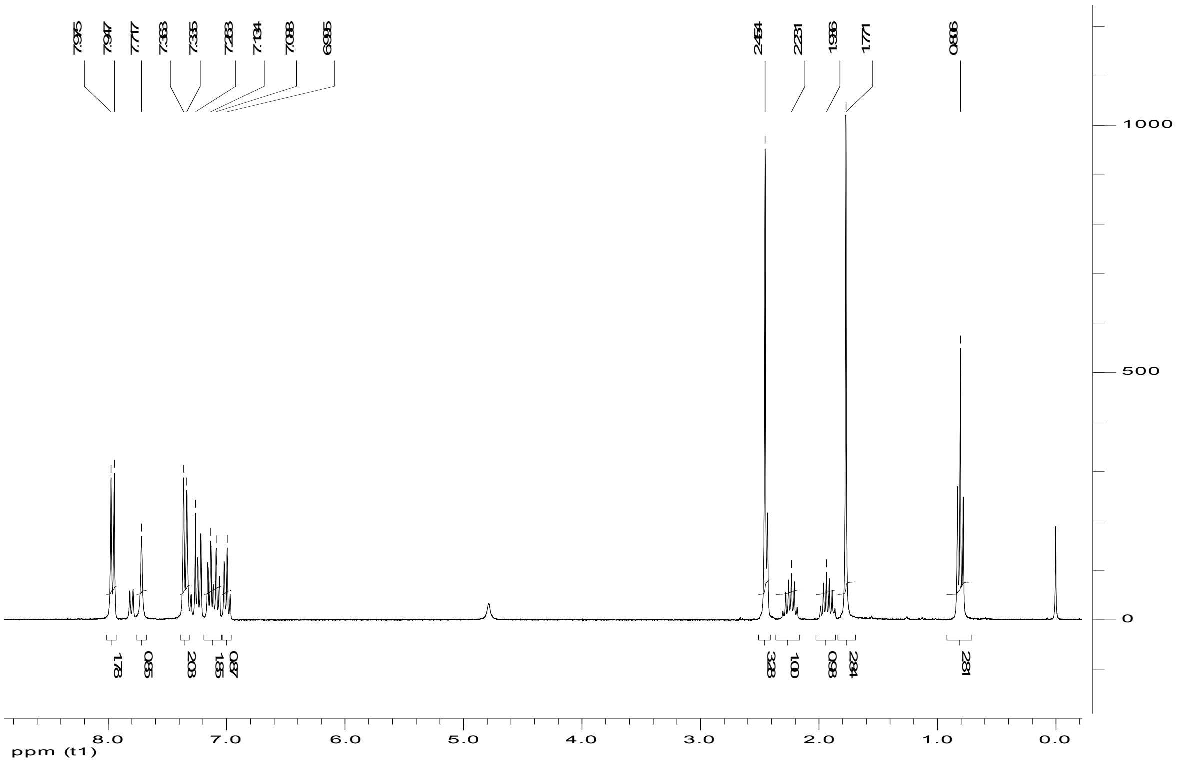

[13]C-NHR:

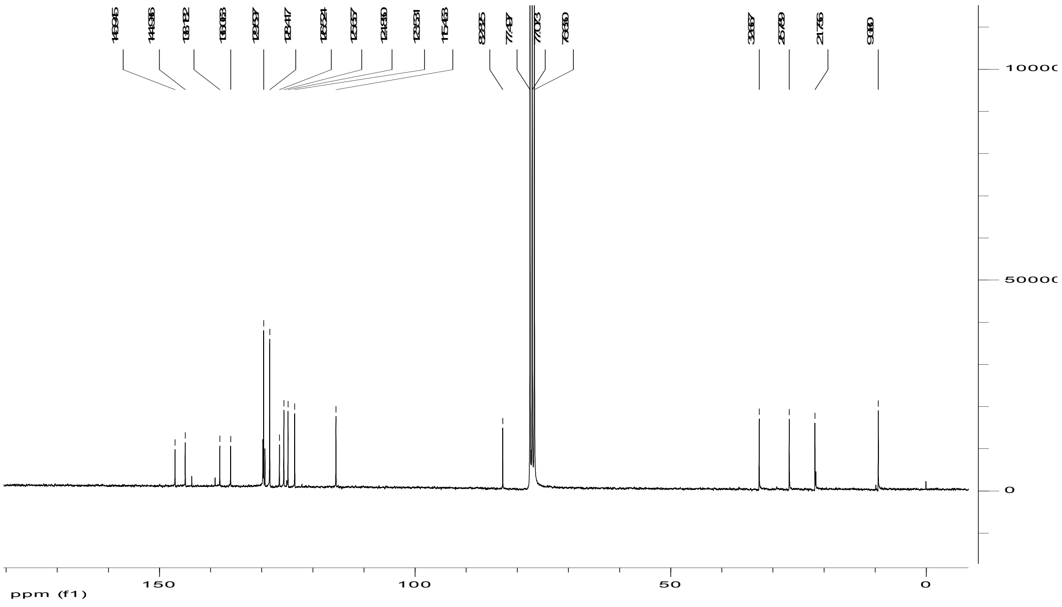

The HRMS was not obtained.

2-propyl-2-methyl-N-[(4-methylphenyl)sulfonyl]-1,3-benzothiazoline-3(2H)-formamide (**3c**)

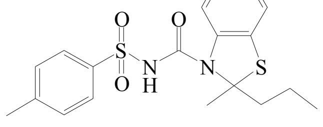

IR:

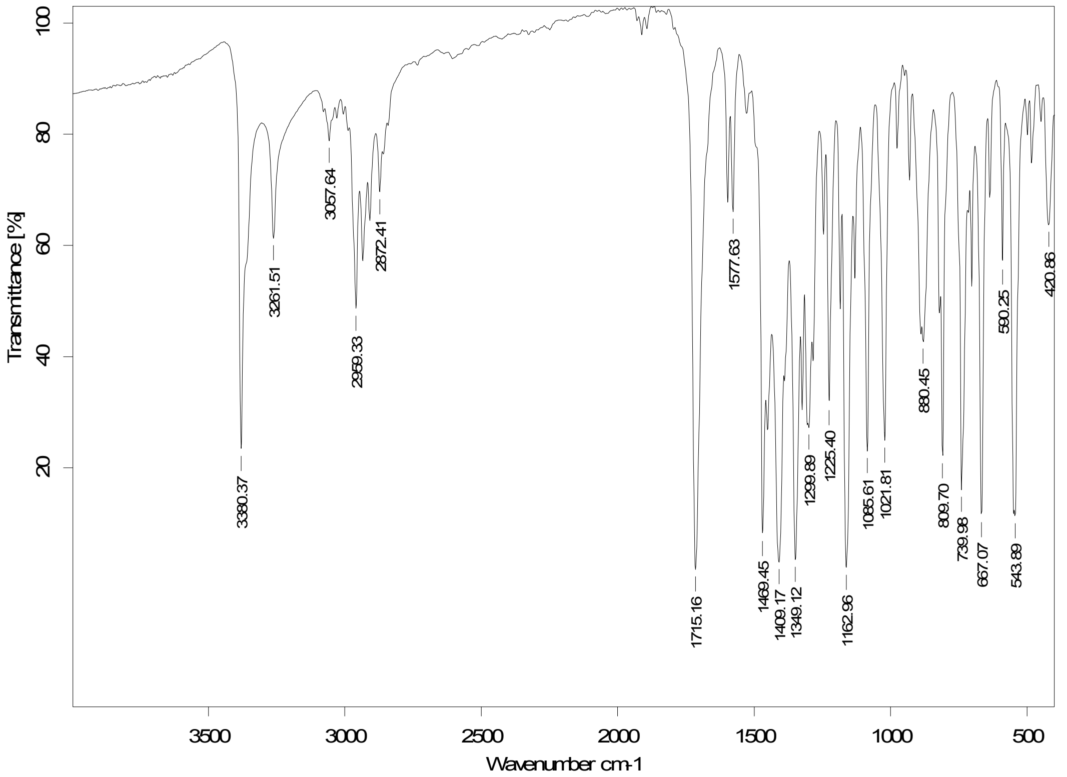

^{1}H-NHR：

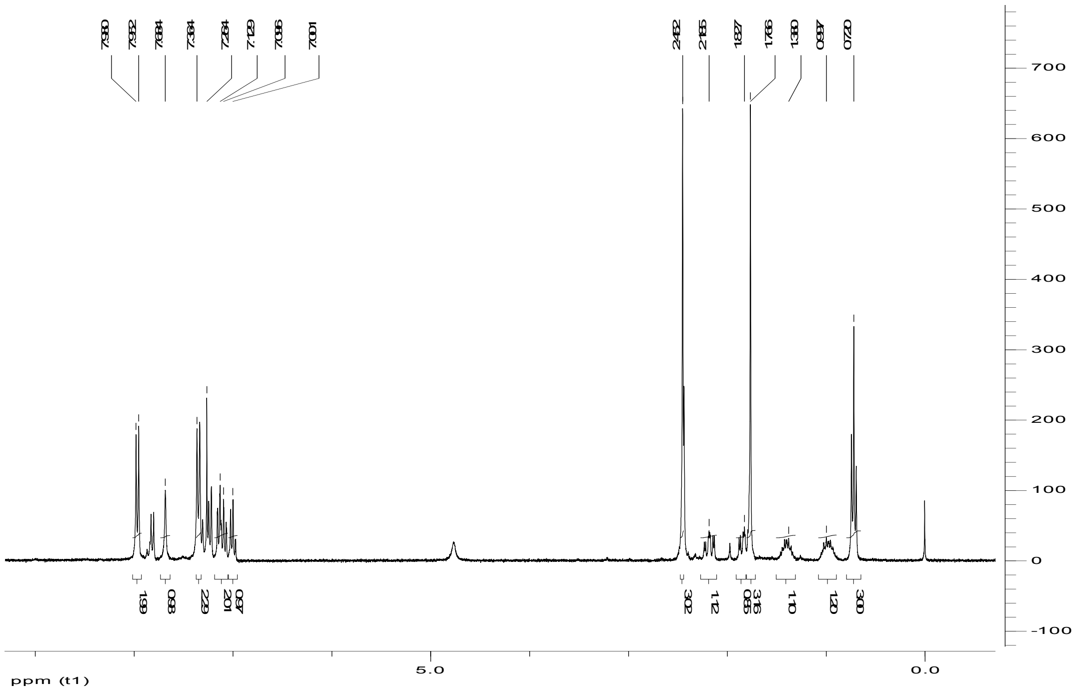

^{13}C-NHR：

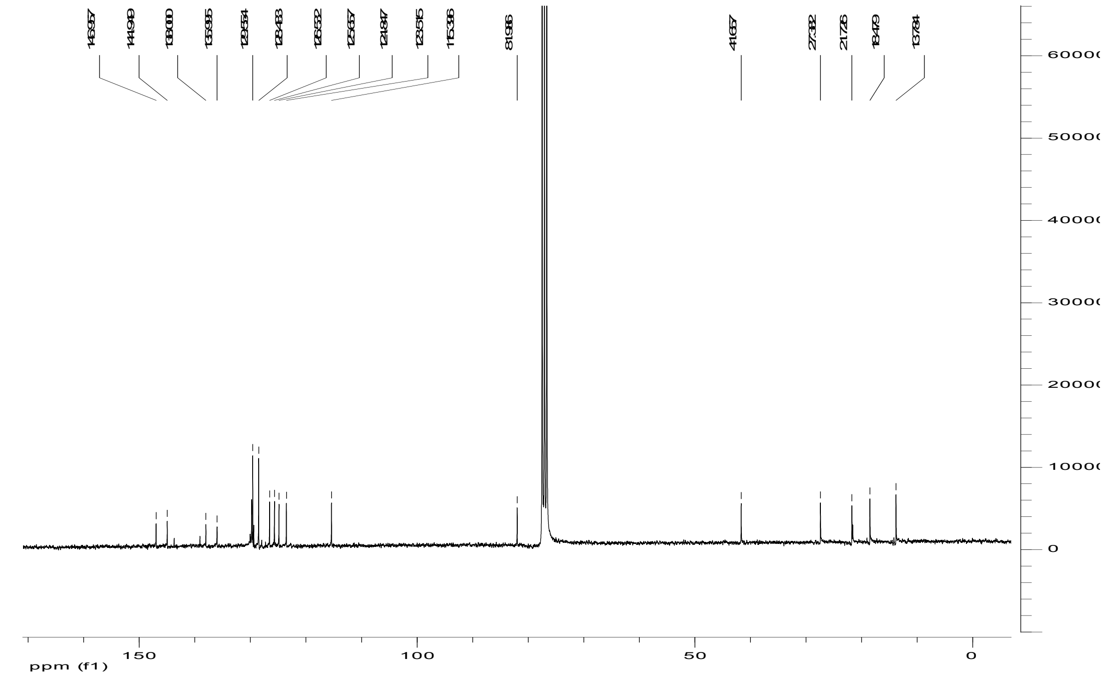
60000
50000
40000
30000
20000
10000
0
150
100
50
0
ppm (f1)

HRMS:

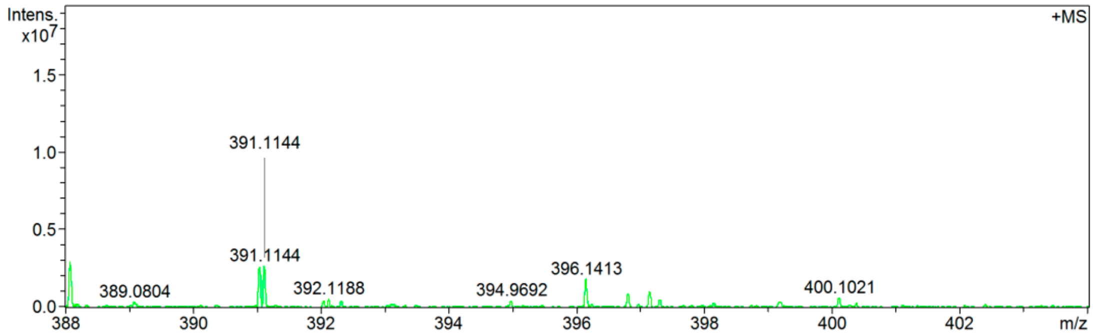
Intens.
x10^7
1.5
1.0
0.5
0.0
+MS
391.1144
391.1144
389.0804
392.1188
394.9692
396.1413
400.1021
388
390
392
394
396
398
400
402
m/z

2-isopropyl-2-methyl-N-[(4-methylphenyl)sulfonyl]-1,3-benzothiazoline-3(2H)- formamide (**3d**)

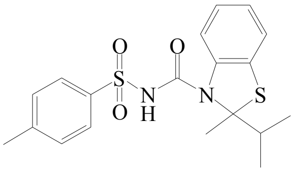

IR:

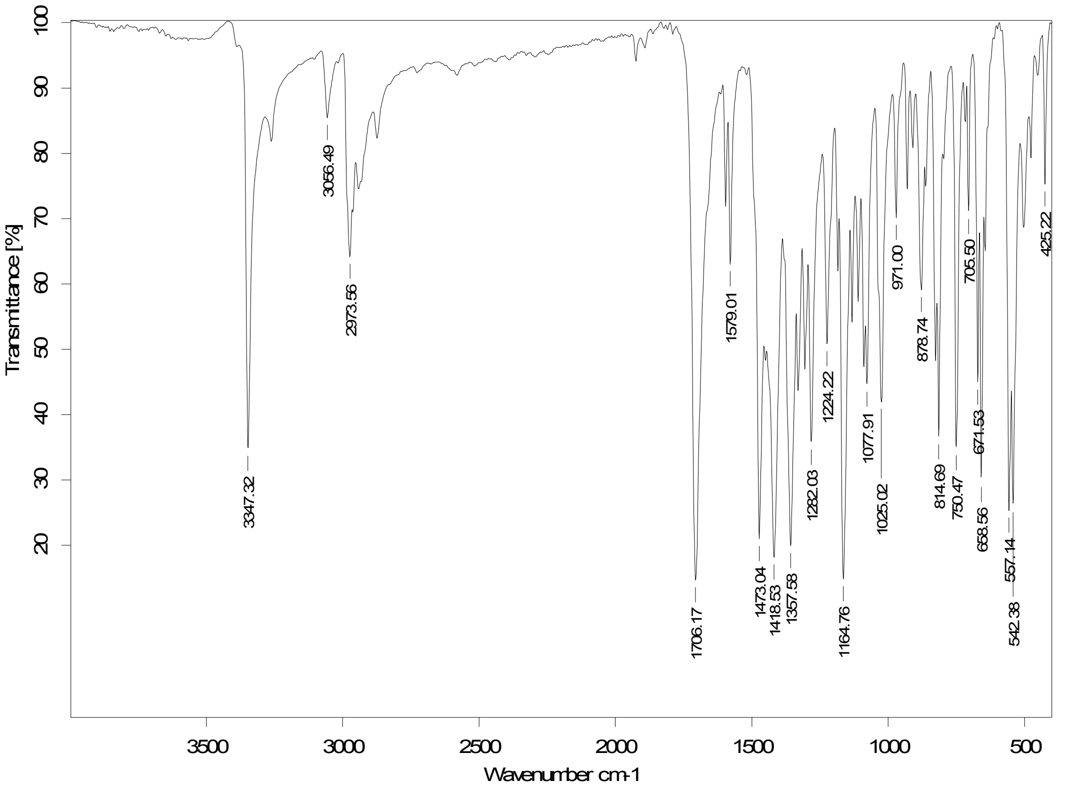

[1]H-NHR:

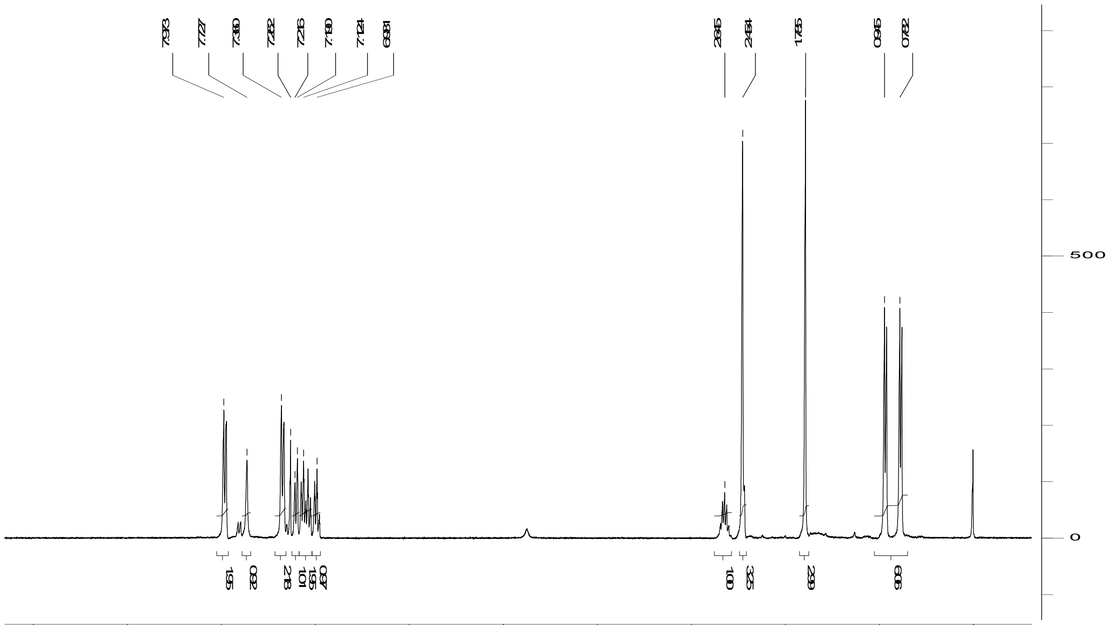

^{13}C-NHR：

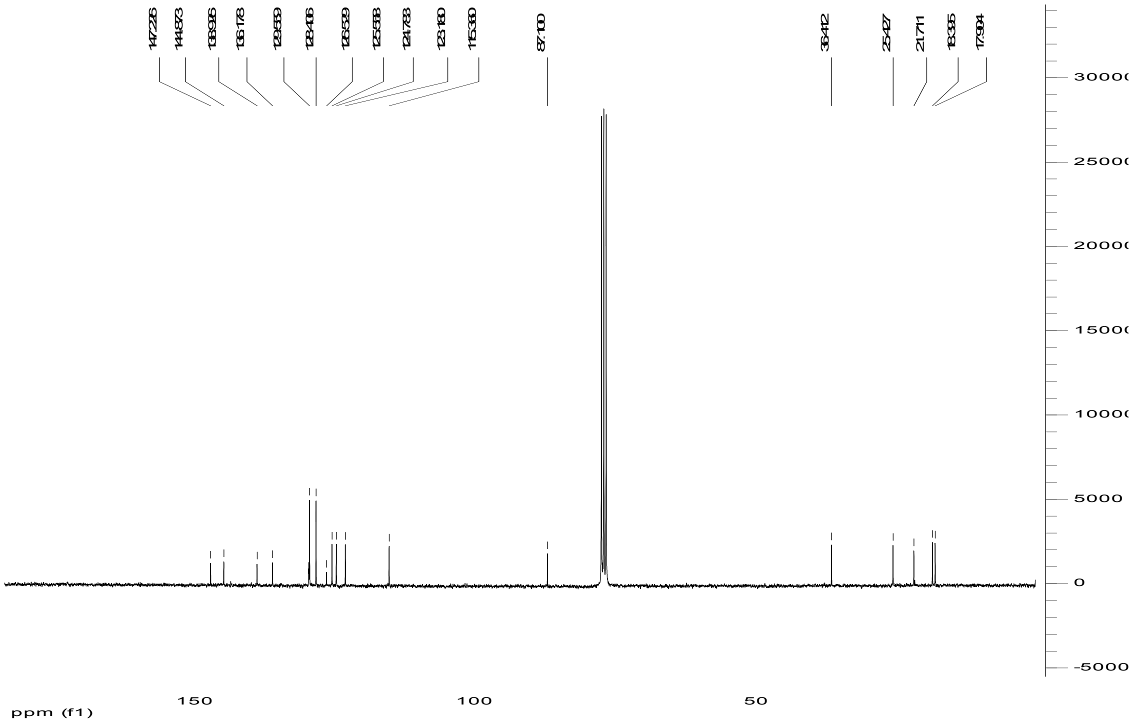

HRMS:

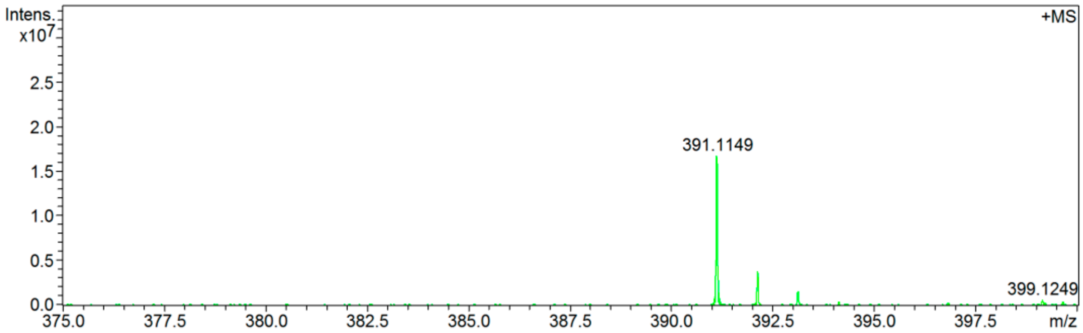

2-acetonyl-2-methyl-N-[(4-methylphenyl)sulfonyl]-1,3-benzothiazoline-3(2H)-for mamide (**3e**)

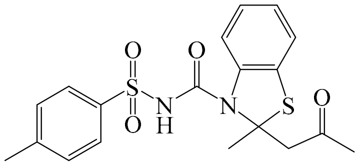

IR:

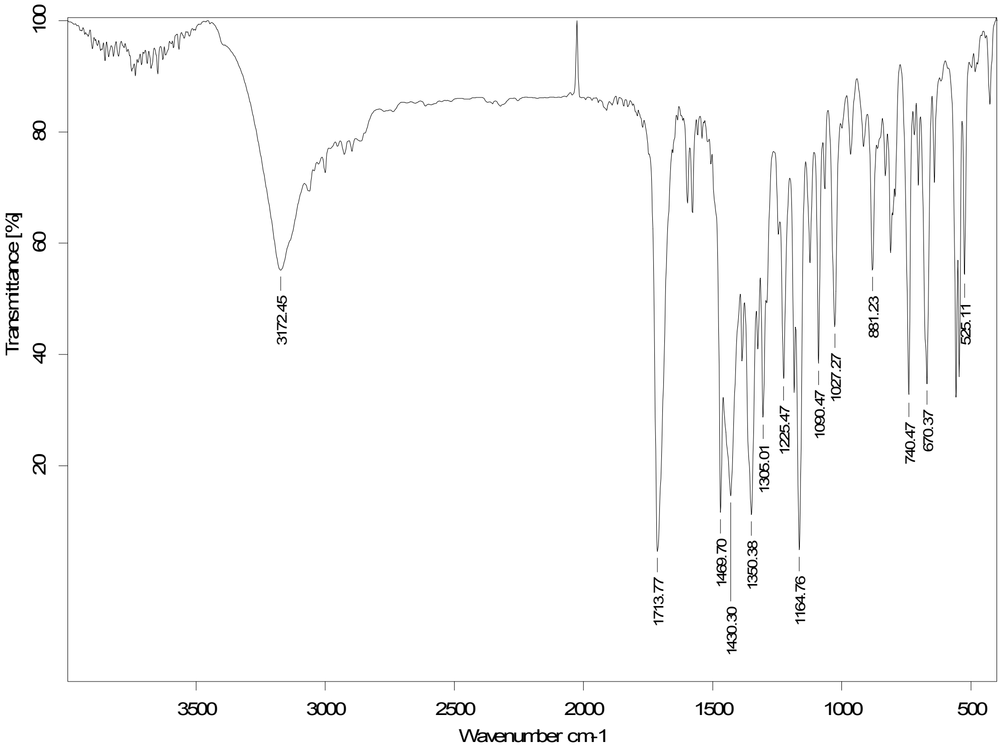

[1]H-NHR：

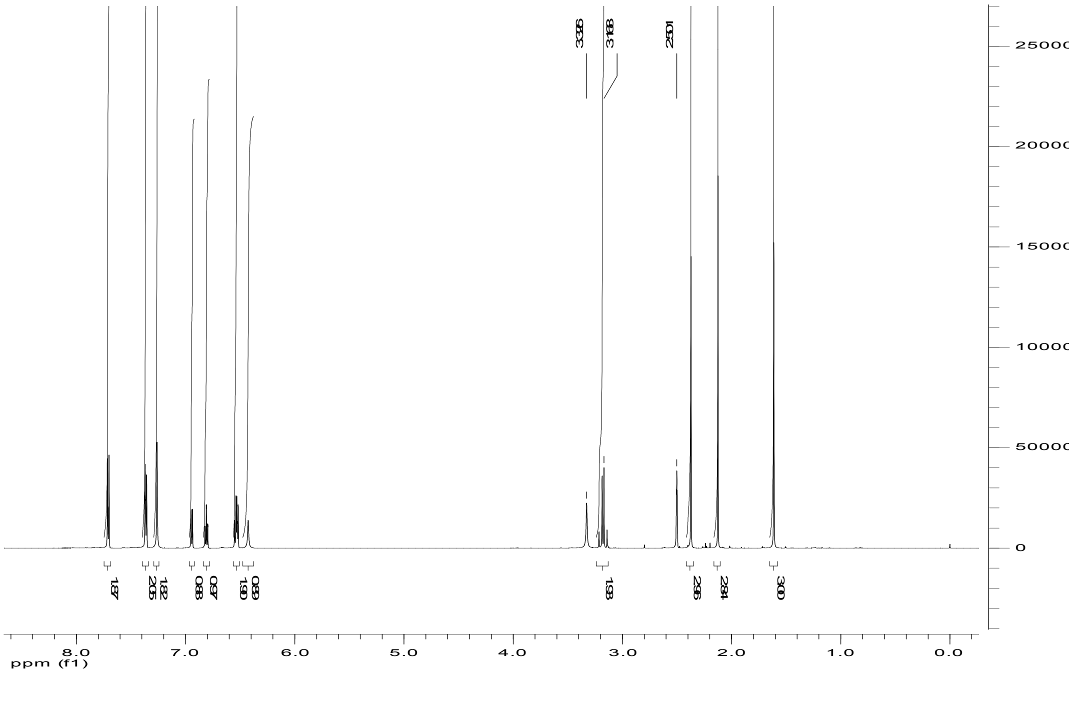

^{13}C-NHR：

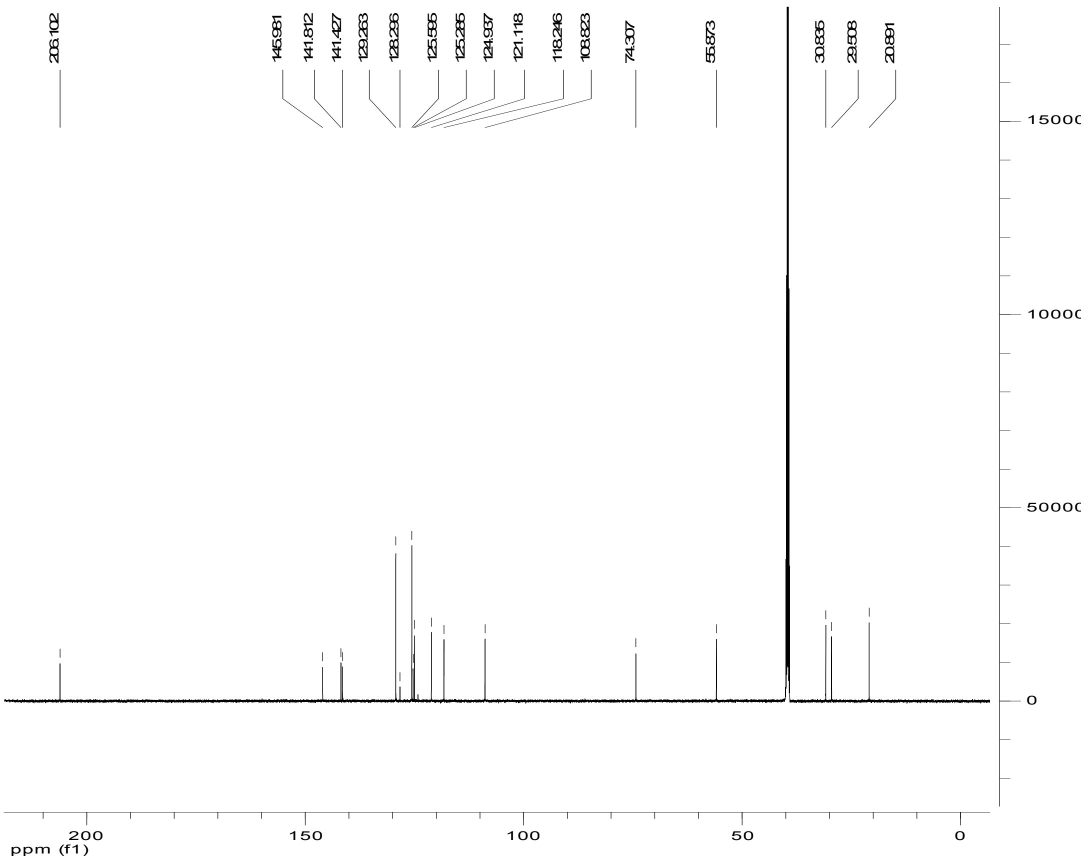
206.102
145.981
141.812
141.427
129.263
128.236
125.595
125.285
124.937
121.118
118.246
108.823
74.307
55.873
30.835
29.508
20.891
15000
10000
50000
0
200
150
100
50
0
ppm (f1)

HRMS:

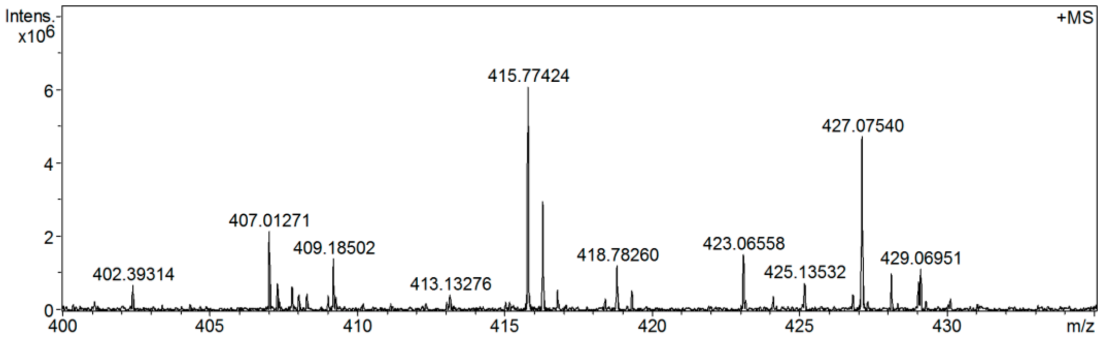
Intens.
x10^6
+MS
415.77424
427.07540
407.01271
409.18502
402.39314
413.13276
418.78260
423.06558
425.13532
429.06951
6
4
2
0
400
405
410
415
420
425
430
m/z

2-2-methyl-N-butyl-[(4-methylphenyl)sulfonyl]-1,3-benzothiazoline-3-(2H)-formamide (**3f**)

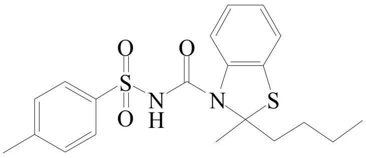

IR:

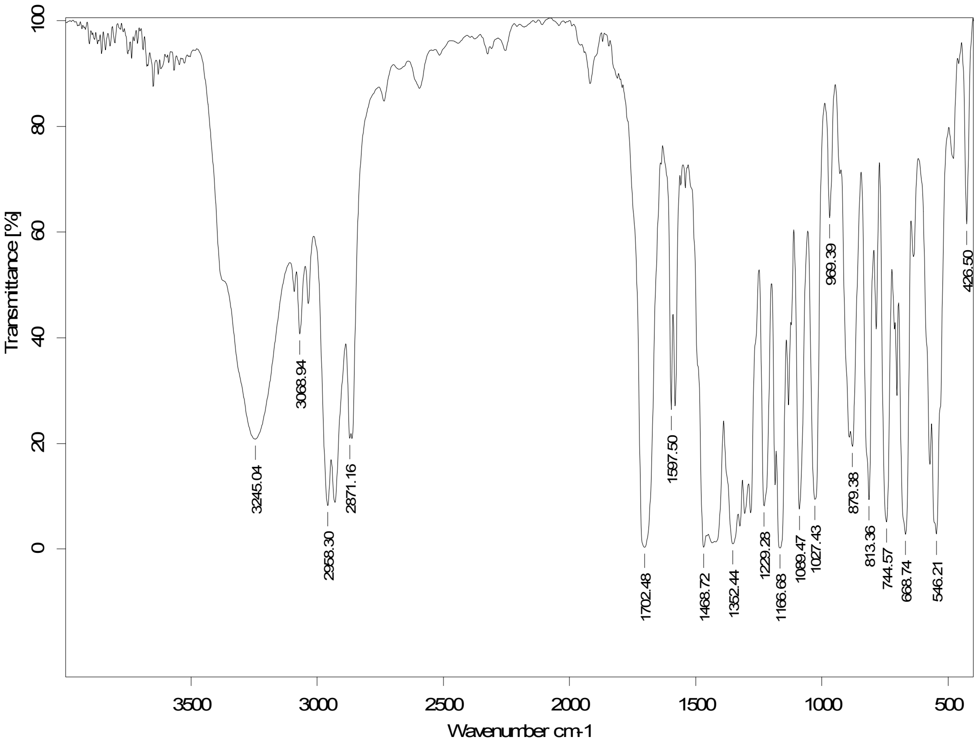

¹H-NHR:

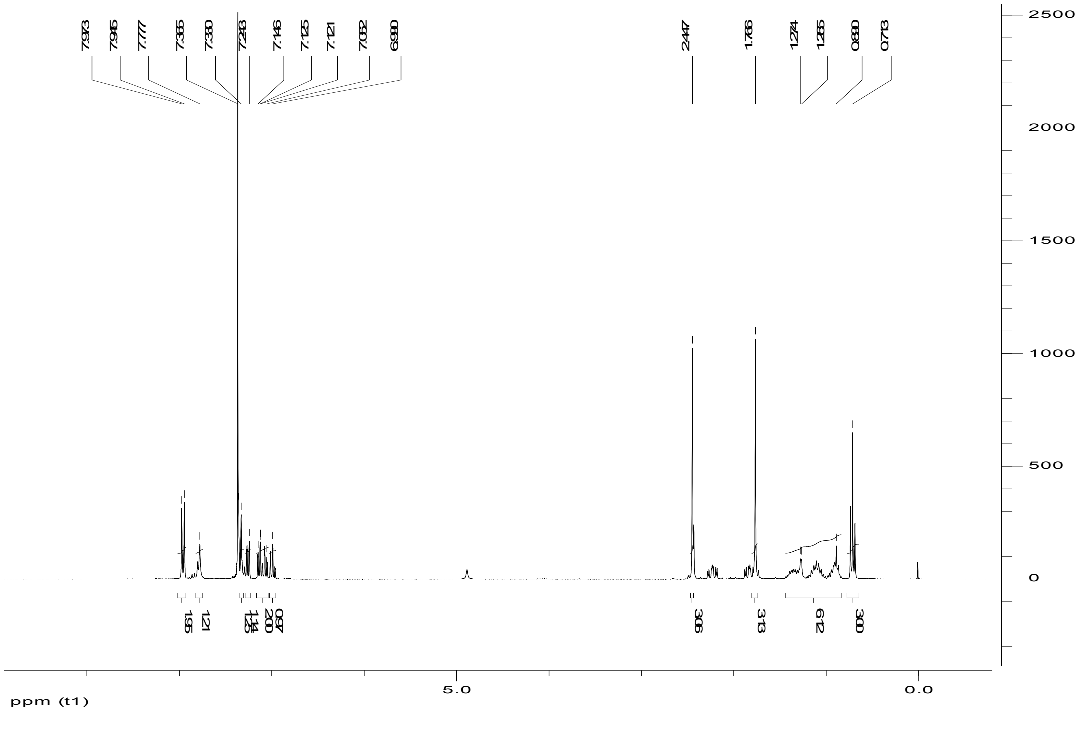

^{13}C-NHR:

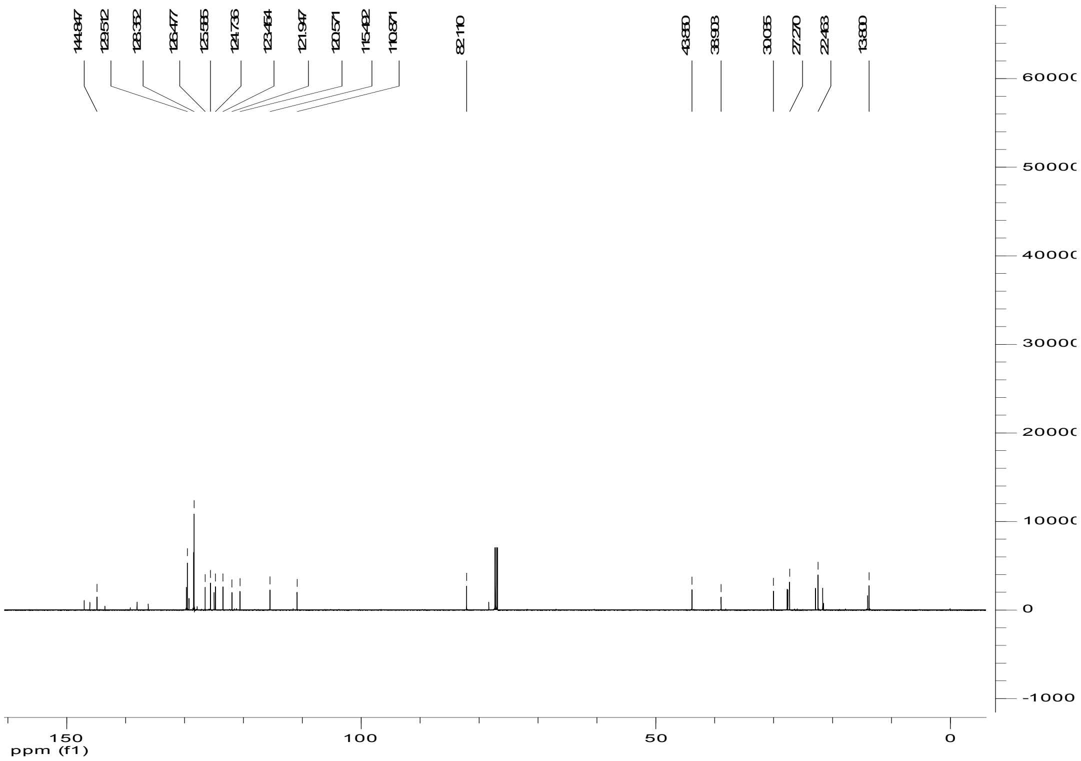
144.847
129.512
128.352
126.477
125.565
124.736
123.464
121.947
120.571
115.482
110.871
82.110
43.880
38.903
30.085
27.270
22.463
13.800
60000
50000
40000
30000
20000
10000
0
-1000
150
100
50
0
ppm (f1)

HRMS:

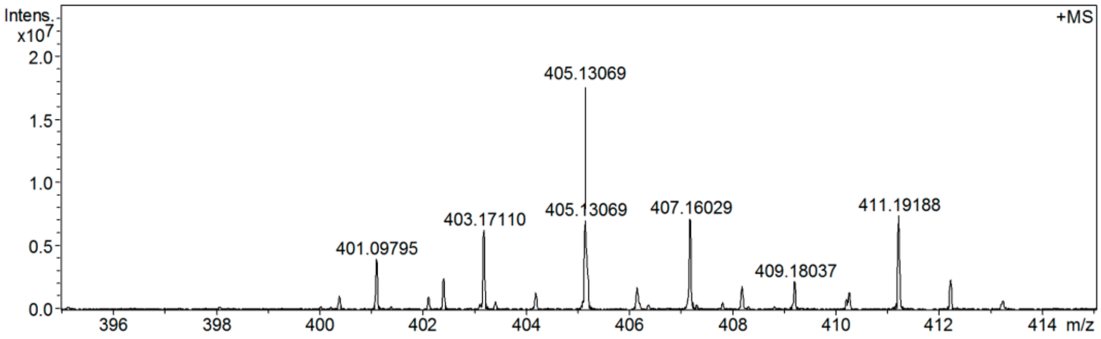
Intens.
x10^7
+MS
405.13069
401.09795
403.17110
405.13069
407.16029
409.18037
411.19188
m/z

2-isobutyl-2-methyl-N-[(4-methylphenyl)sulfonyl]-1,3-benzothiazoline-3-(2H)- formamide (**3g**)

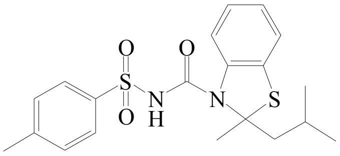

IR:

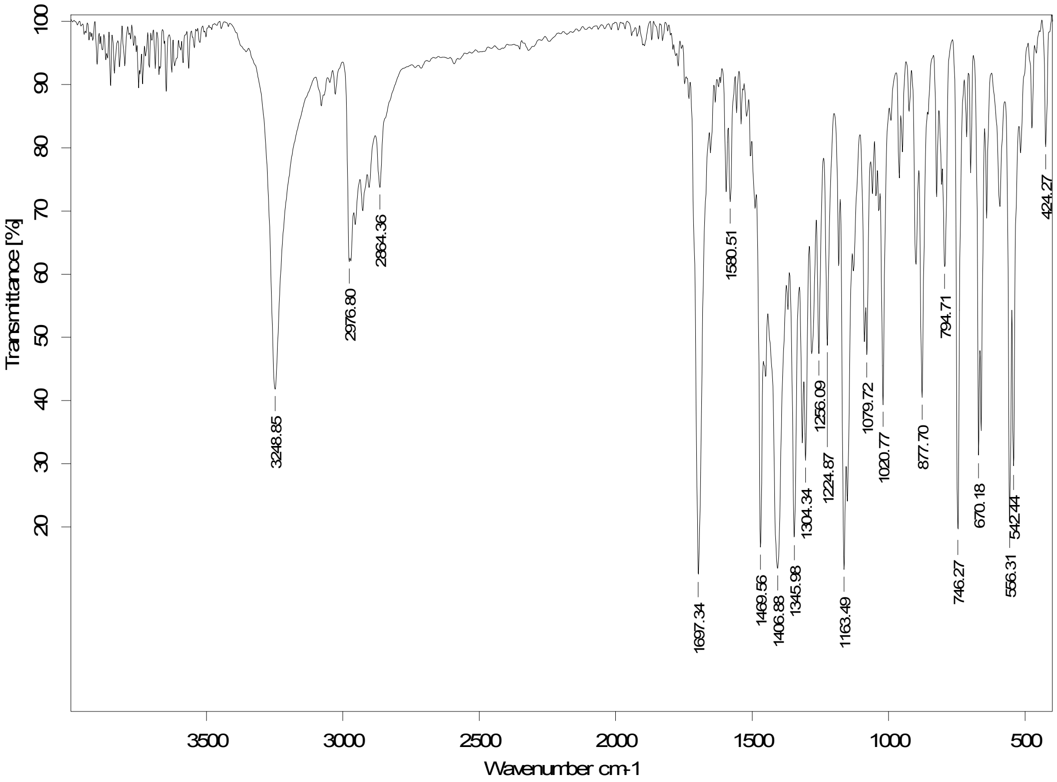

¹H-NHR：

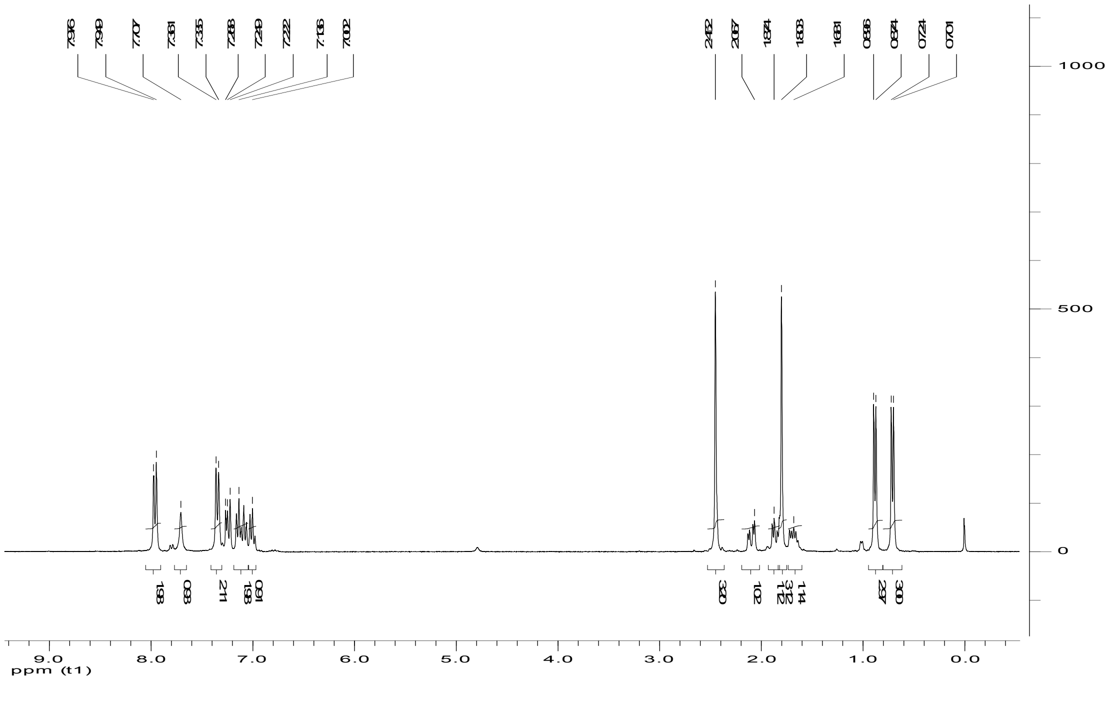

[13]C-NHR：

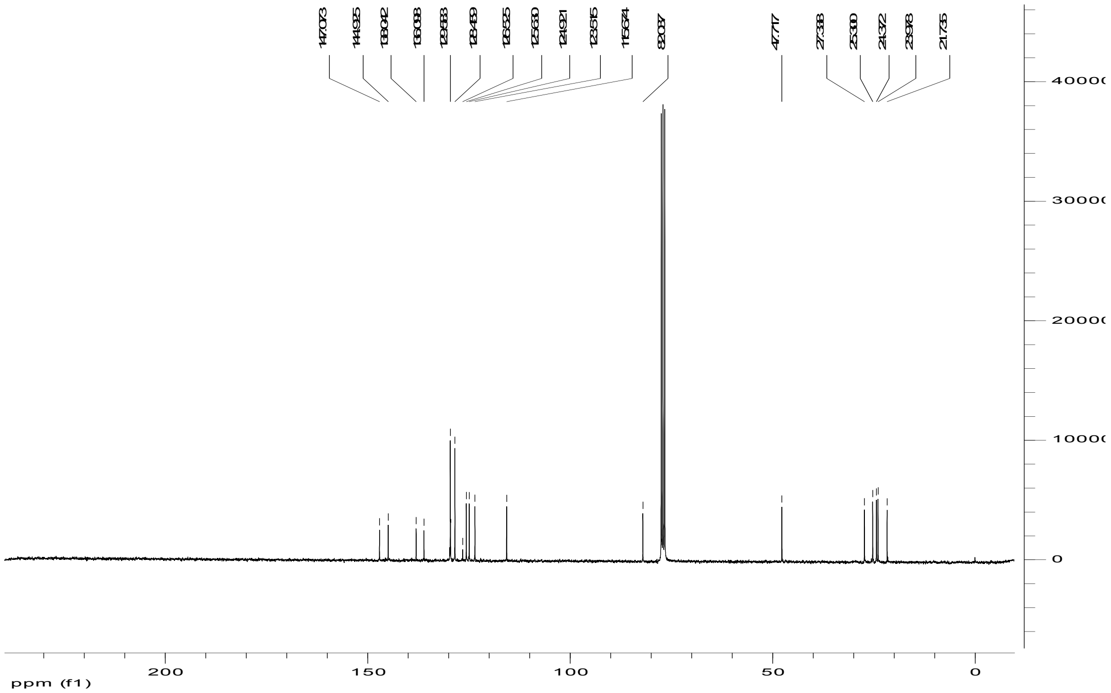

The HRMS was not obtained.

2-tert-butyl-2-methyl-N-[(4-methylphenyl)sulfonyl]-1,3benzothiazoline-3(2H)- formamide (**3h**)

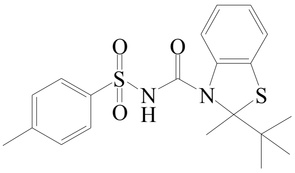

IR

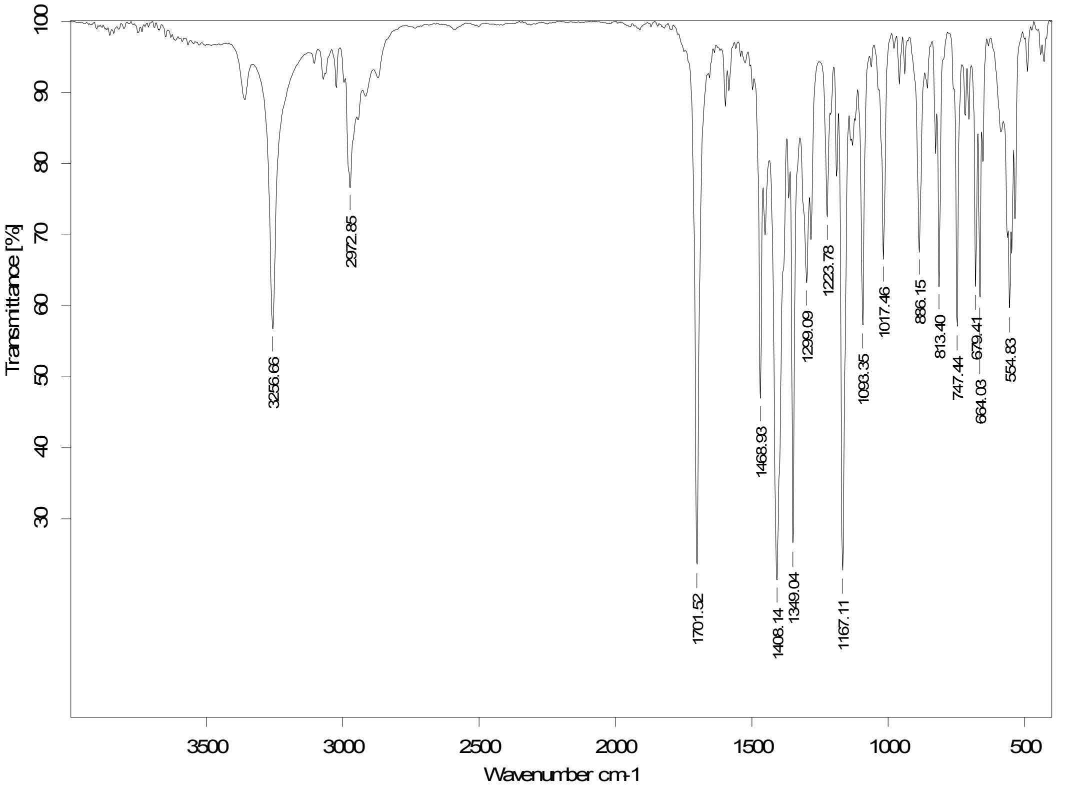

[1]H-NHR：

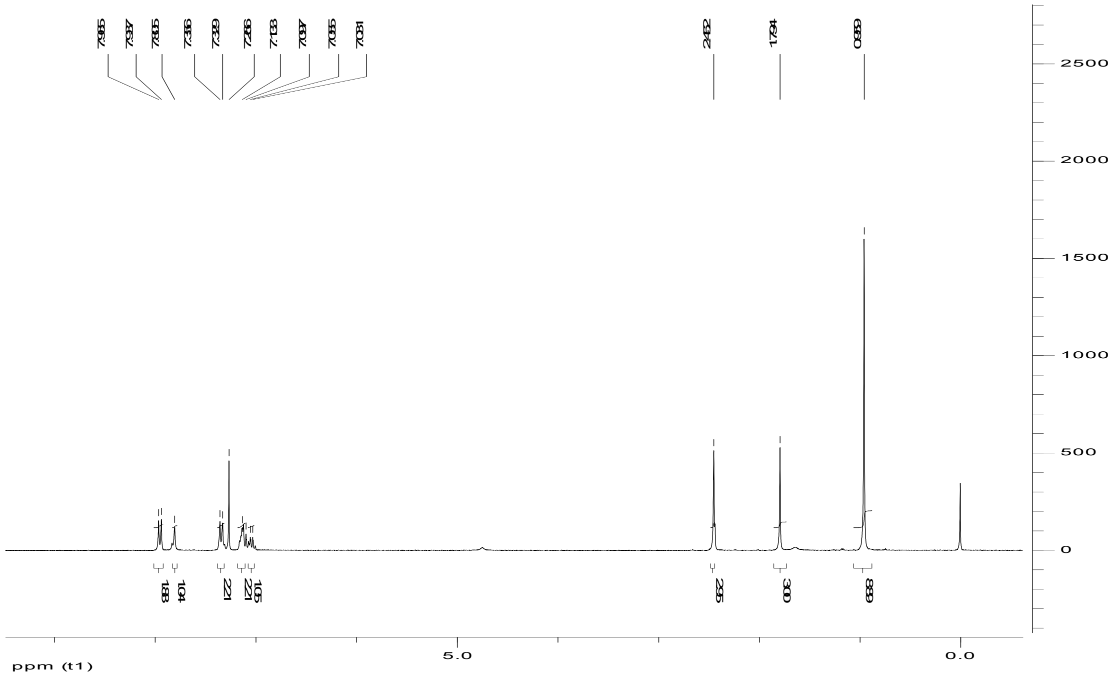

^{13}C-NHR：

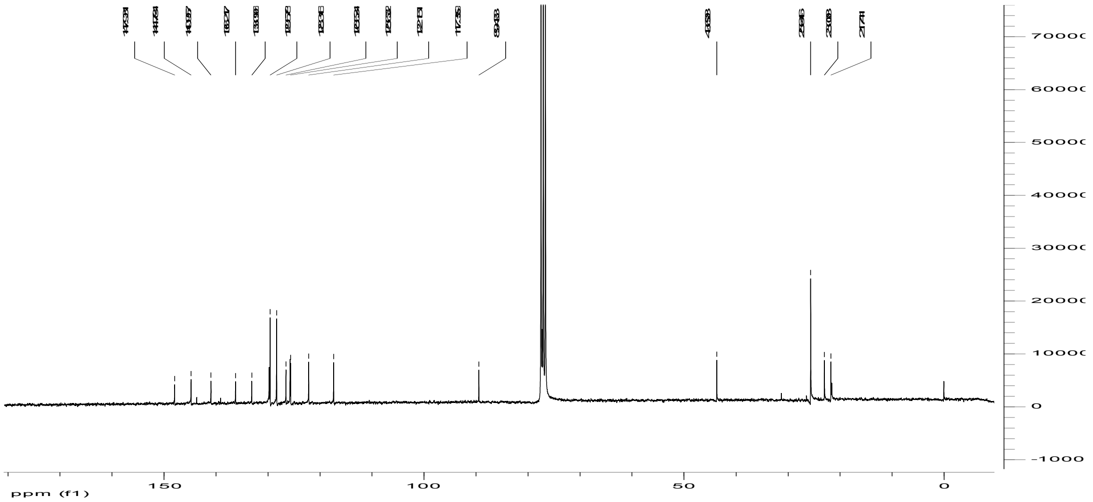

ppm (f1)
150
100
50
0
70000
60000
50000
40000
30000
20000
10000
0
-1000

HRMS:

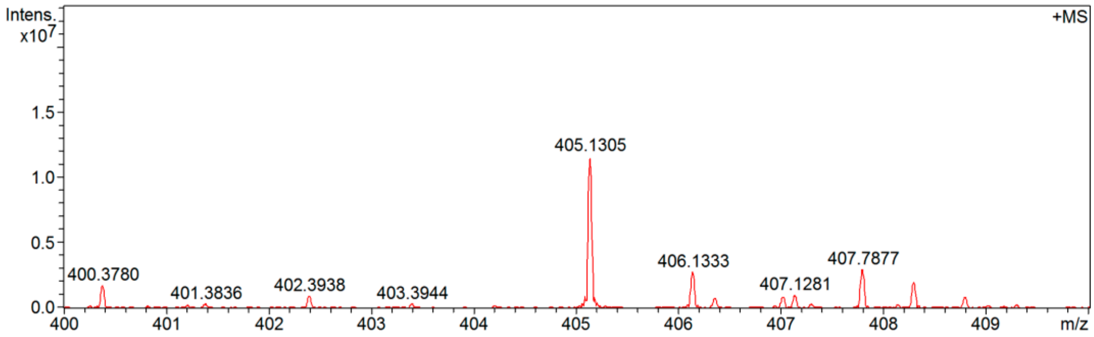

Intens.
x10^7
+MS
1.5
1.0
0.5
0.0
405.1305
400.3780
401.3836
402.3938
403.3944
406.1333
407.1281
407.7877
400
401
402
403
404
405
406
407
408
409
m/z

2-phenylethyl-2-methyl-N-[(4-methylphenyl)sulfonyl]-1,3–benzothiazoline-3(2 H)-formamide (**3i**)

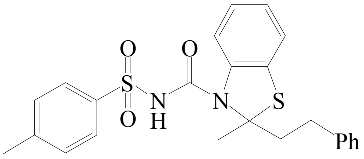

IR:

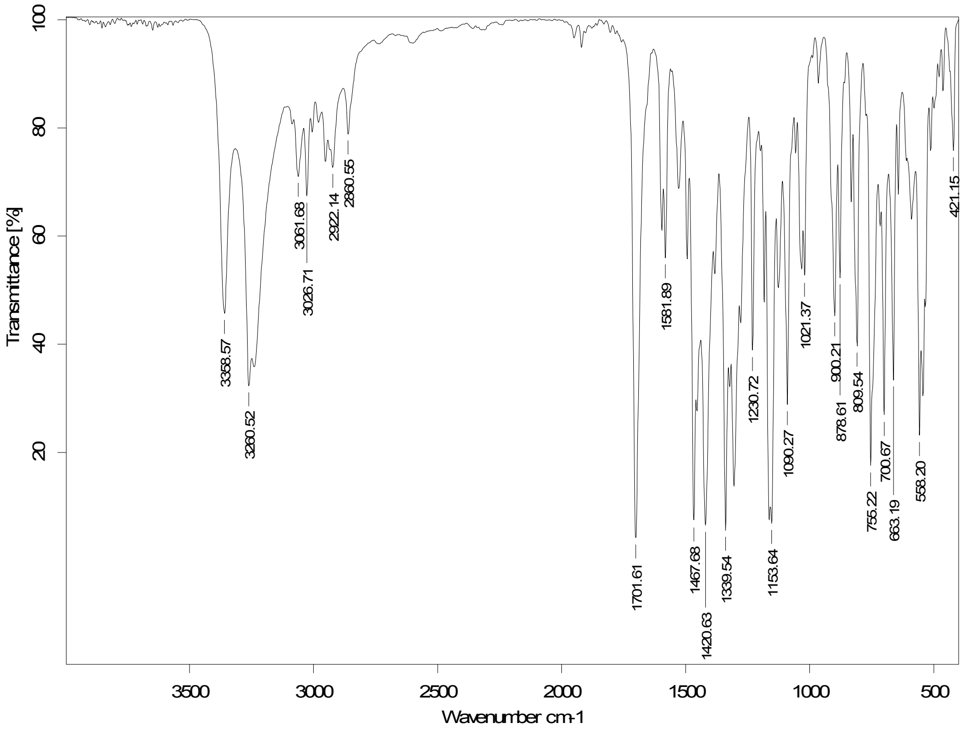

[1]H-NHR：

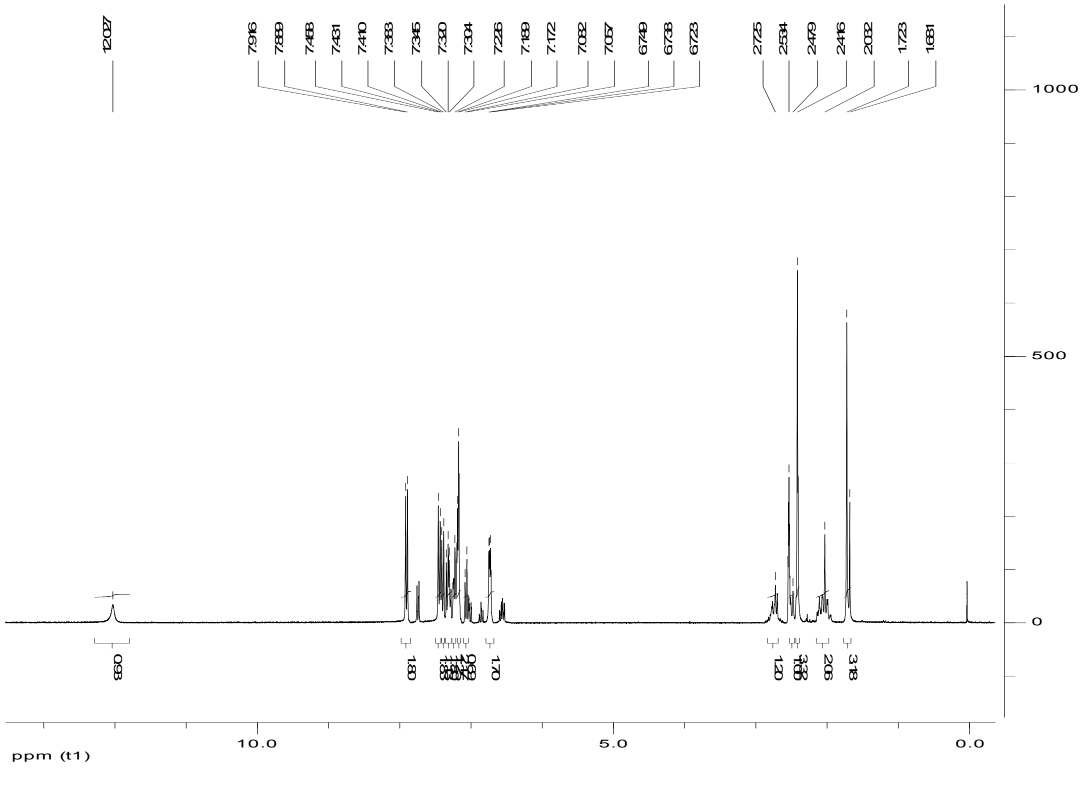

^{13}C-NHR：

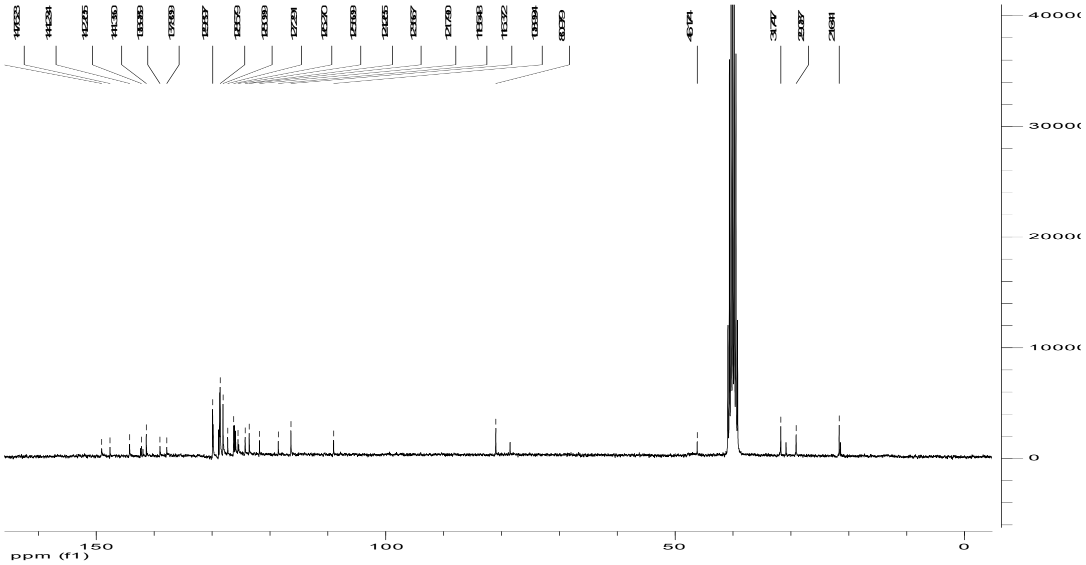

HRMS:

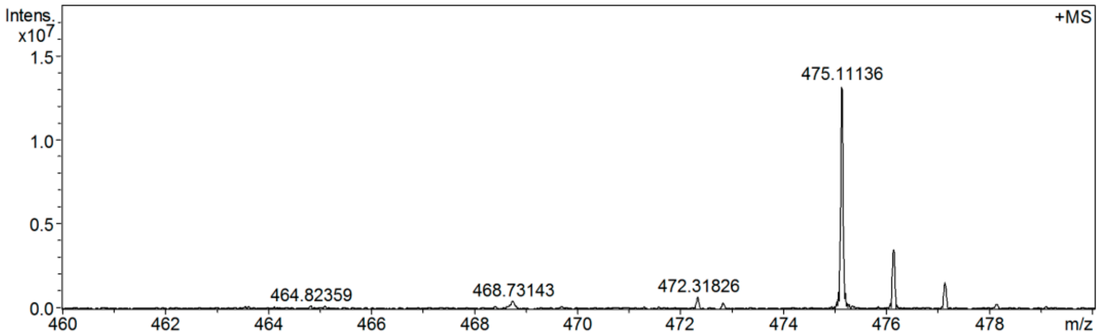

2,2,-diethyl-N-[(4-methylphenyl)sulfonyl]-1,3-benzothiazoline-3(2H)-formamide (**3j**)

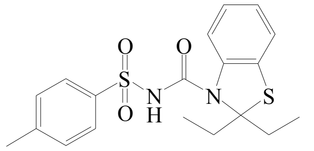

IR:

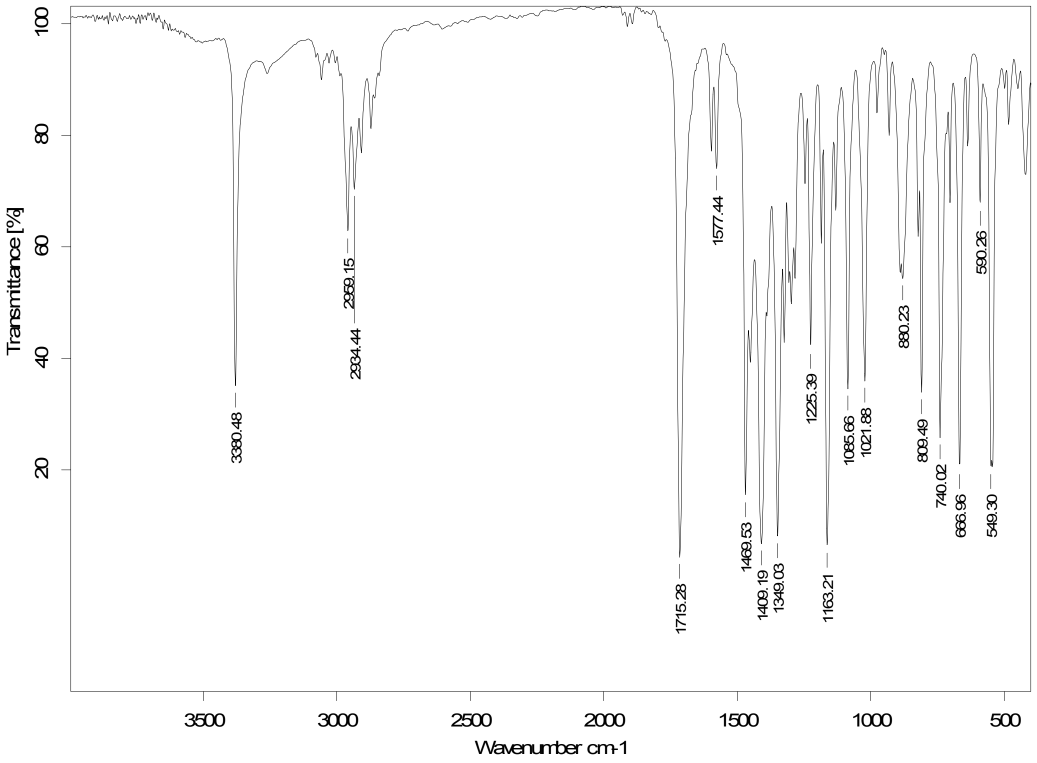

^{1}H-NHR:

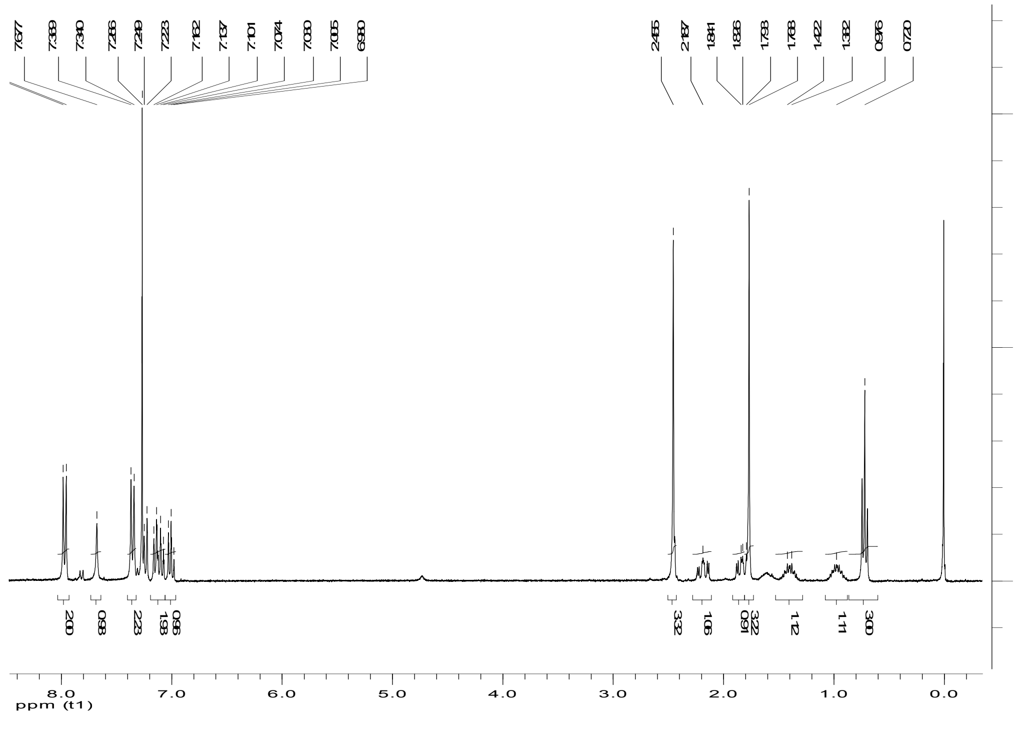

^{13}C-NHR：

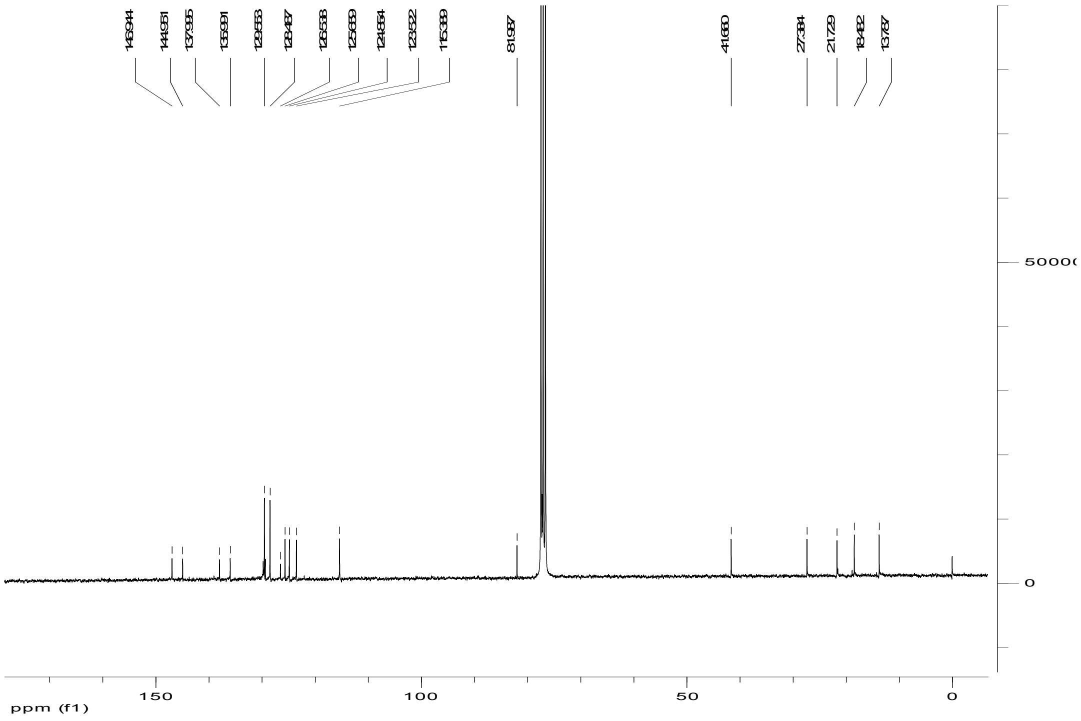

The HRMS was not obtained.

2,2,-dipropyl-N-[(4-methylphenyl)sulfonyl]-1,3-benzothiazoline-3(2H)–formamide (**3k**)

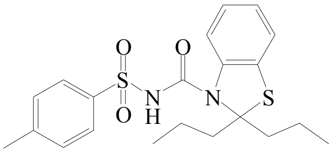

IR:

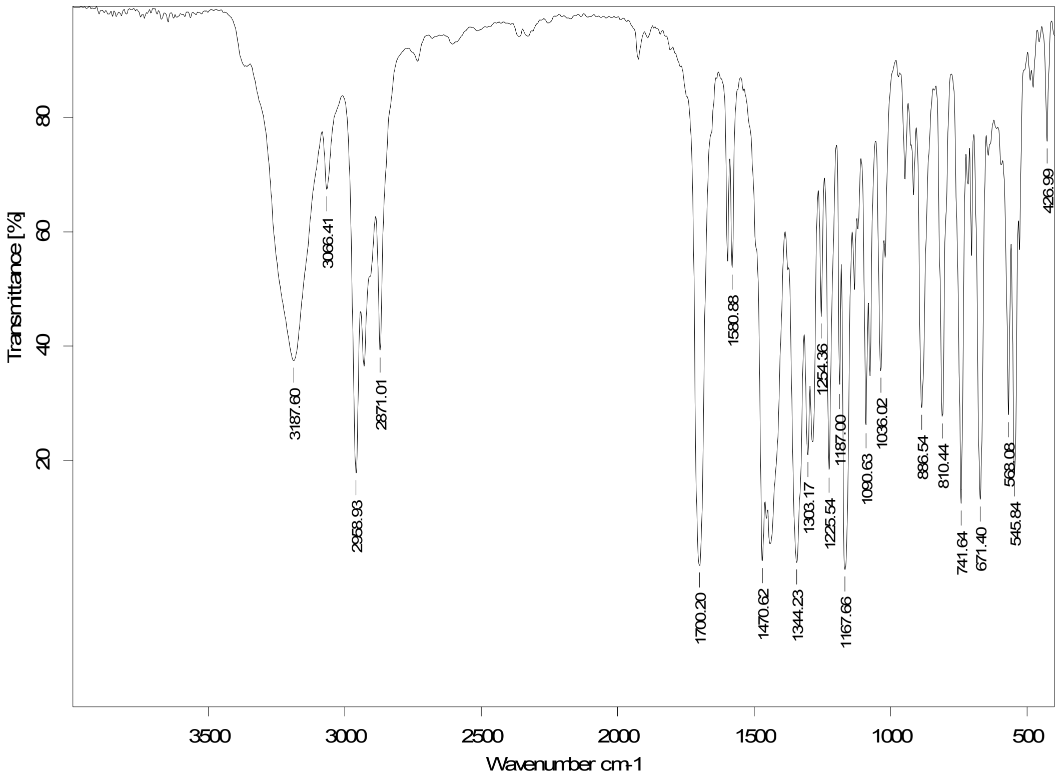

[1]H-NHR：

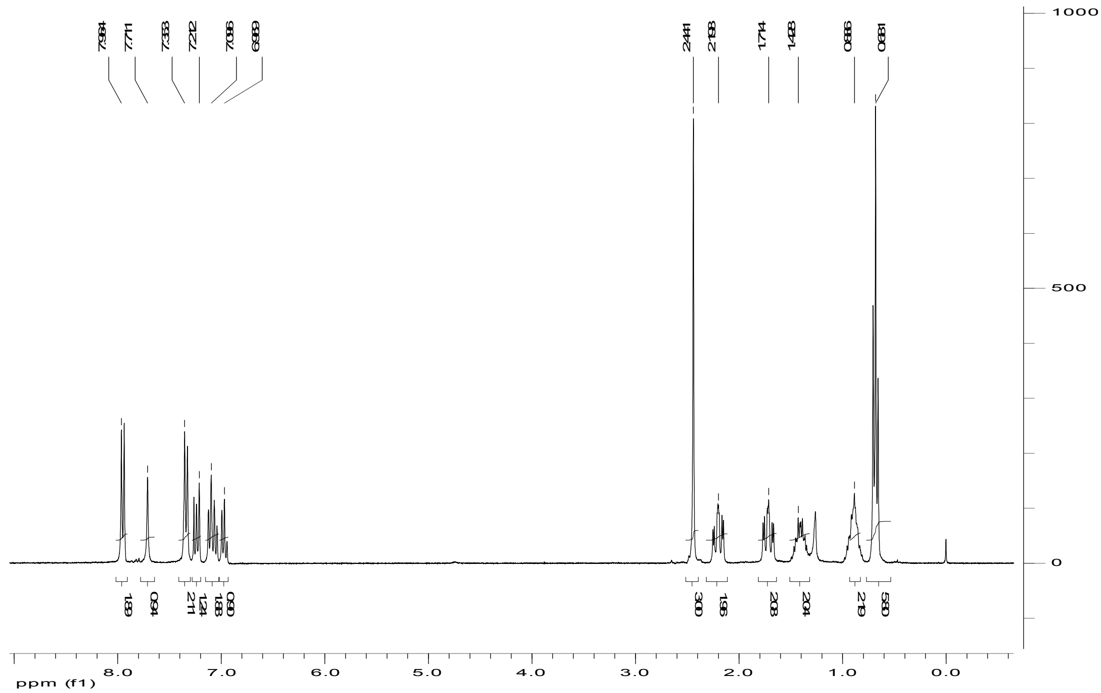

^{13}C-NHR：

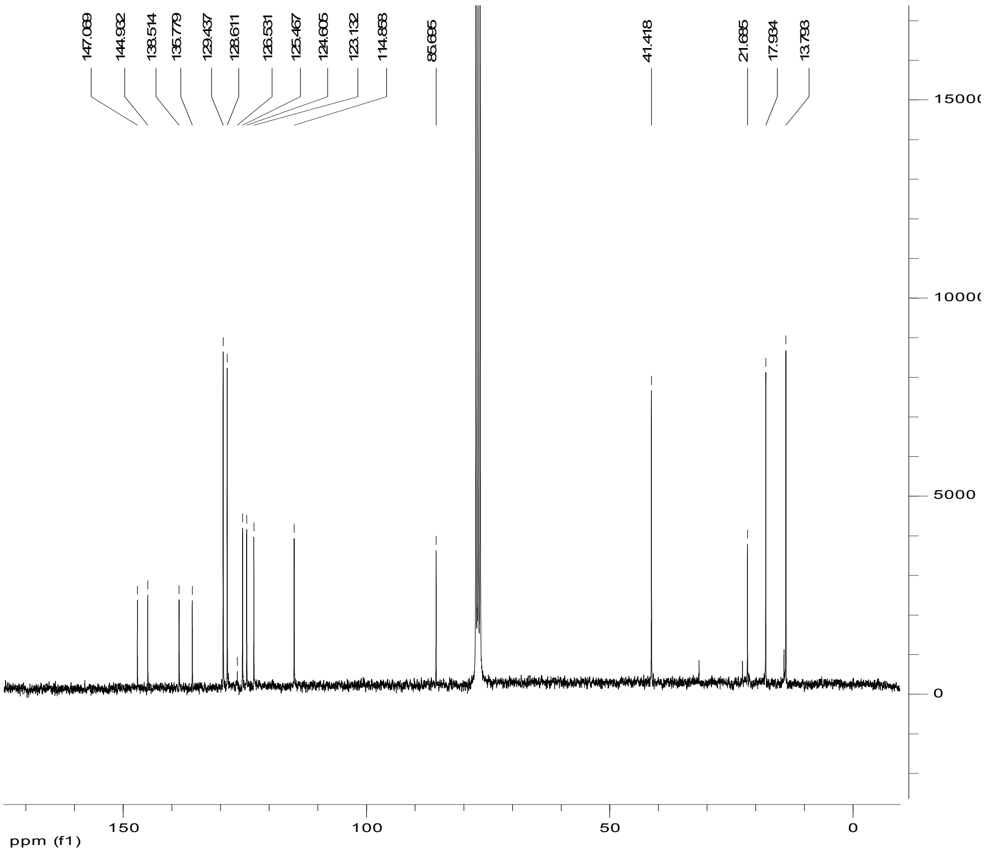

147.069
144.932
138.514
135.779
129.437
128.611
126.531
125.467
124.605
123.132
114.858
85.695
41.418
21.685
17.934
13.793
15000
10000
5000
0
150
100
50
0
ppm (f1)

HRMS:

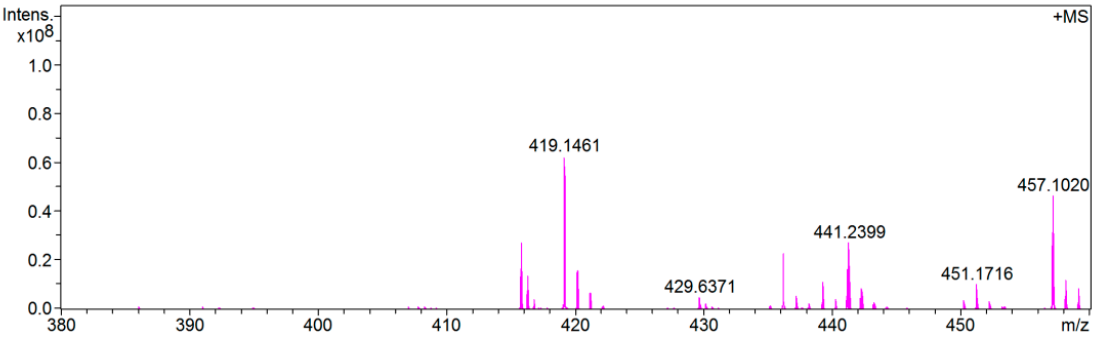

Intens.
x10^8
+MS
1.0
0.8
0.6
0.4
0.2
0.0
419.1461
457.1020
441.2399
451.1716
429.6371
380
390
400
410
420
430
440
450
m/z

N-[(4-methylphenyl)sulfonyl]-3h-screw[1,3-benzothiazoline-2,1'-cyclopentane]   -3-formamide (**3l**)

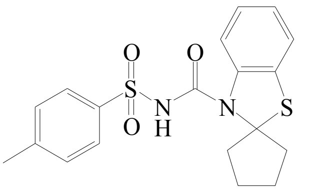

IR:

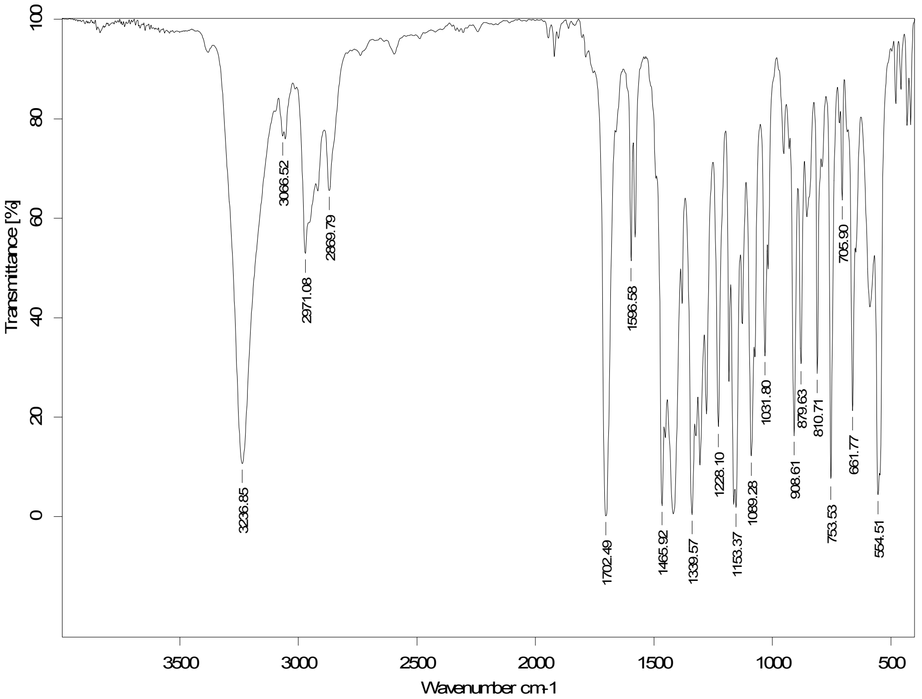

[1]H-NHR：

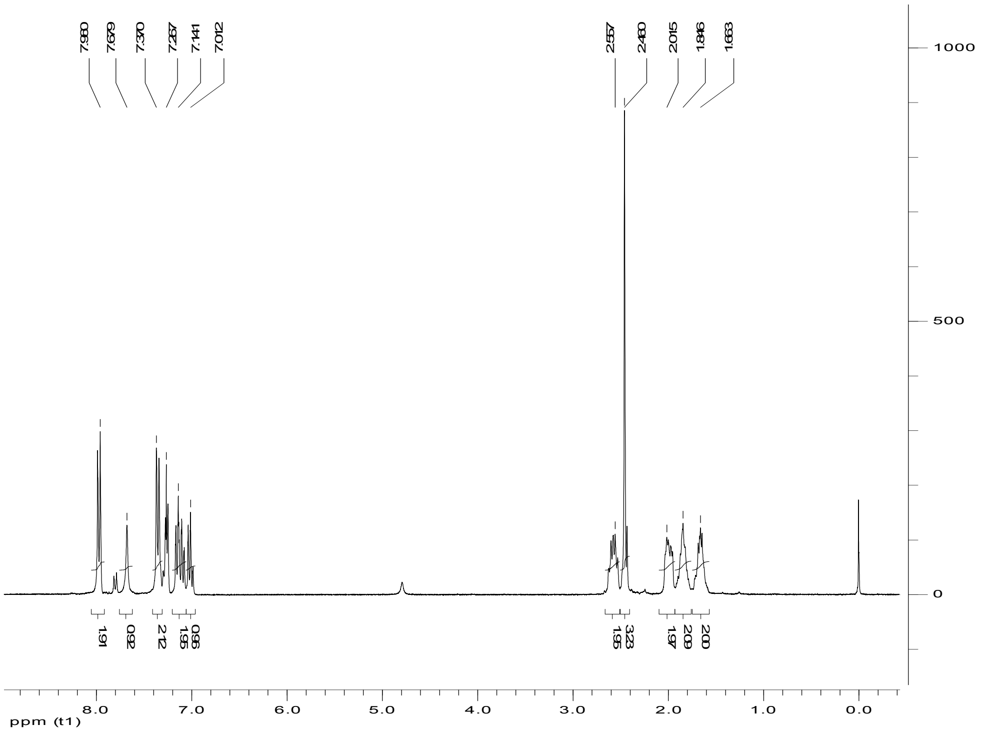

^{13}C-NHR：

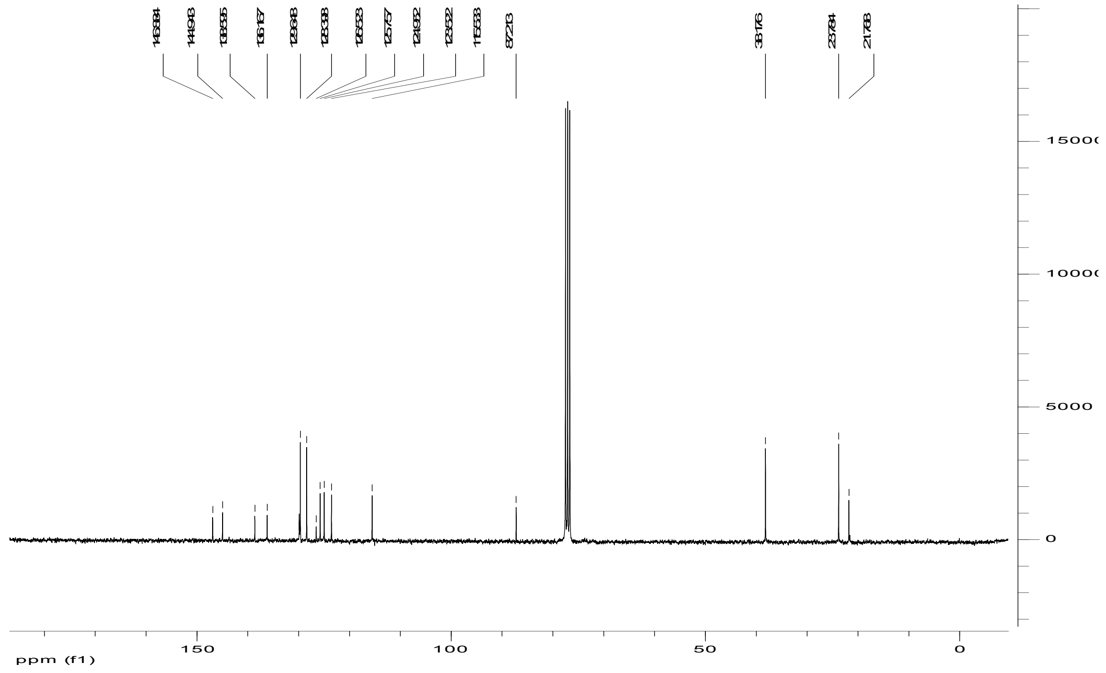

The HRMS was not obtained.

N-[(4-methyl-phenyl)sulfonyl]-3h-screw[1,3-benzothiazoline-2,1'-cyclohexane]-3-formamide (**3m**)

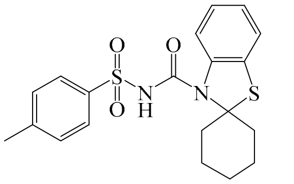

IR:

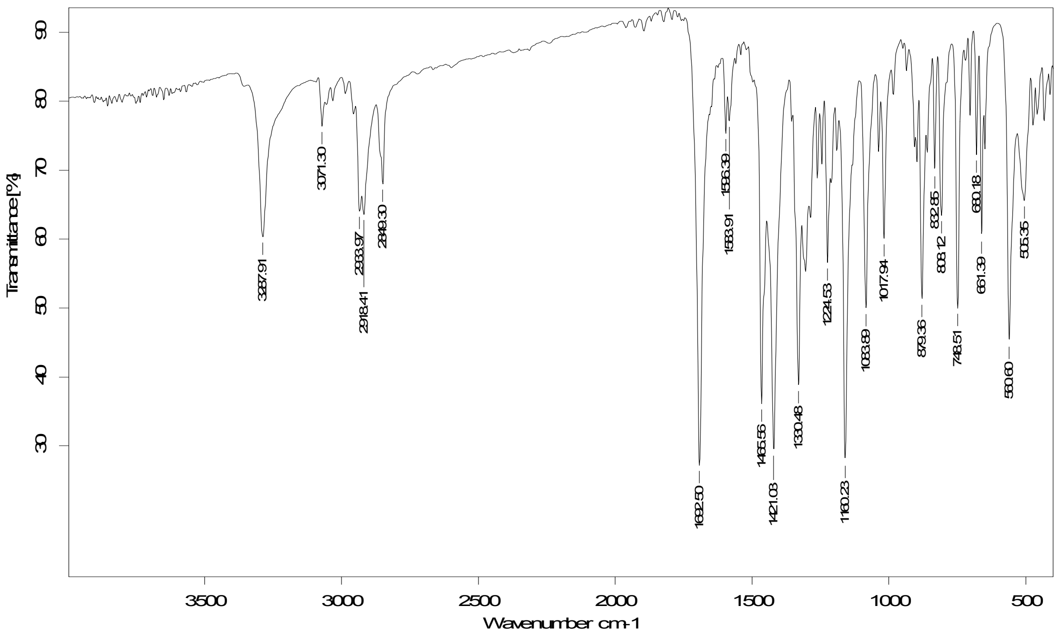

[1]H-NHR:

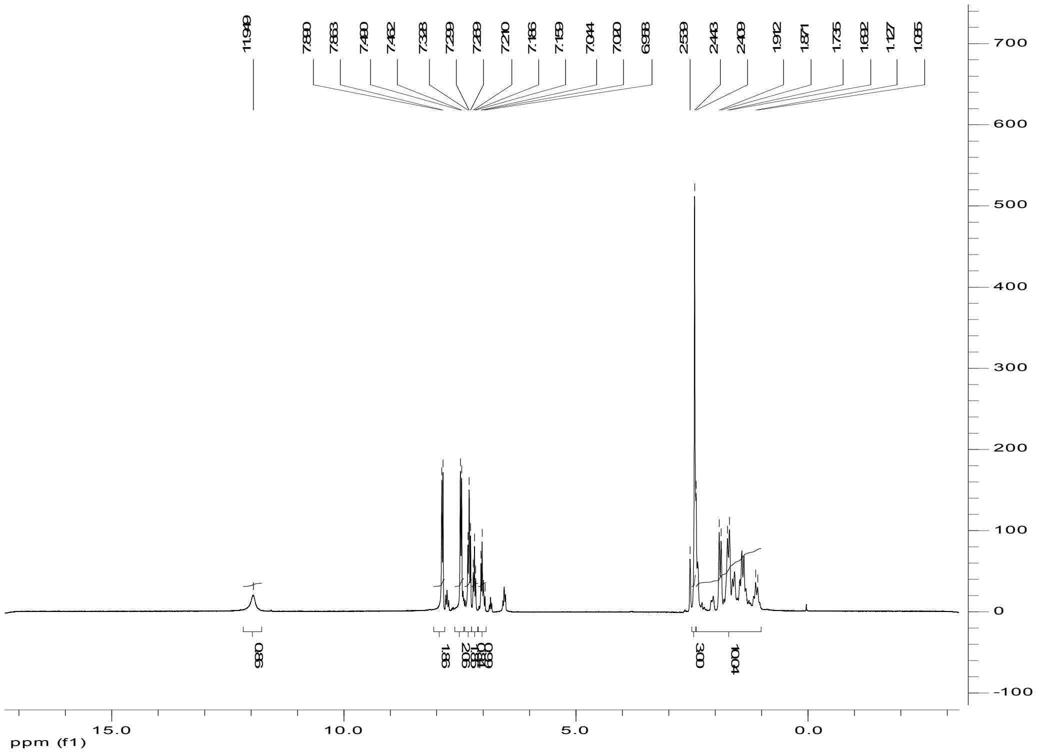

^{13}C-NHR:

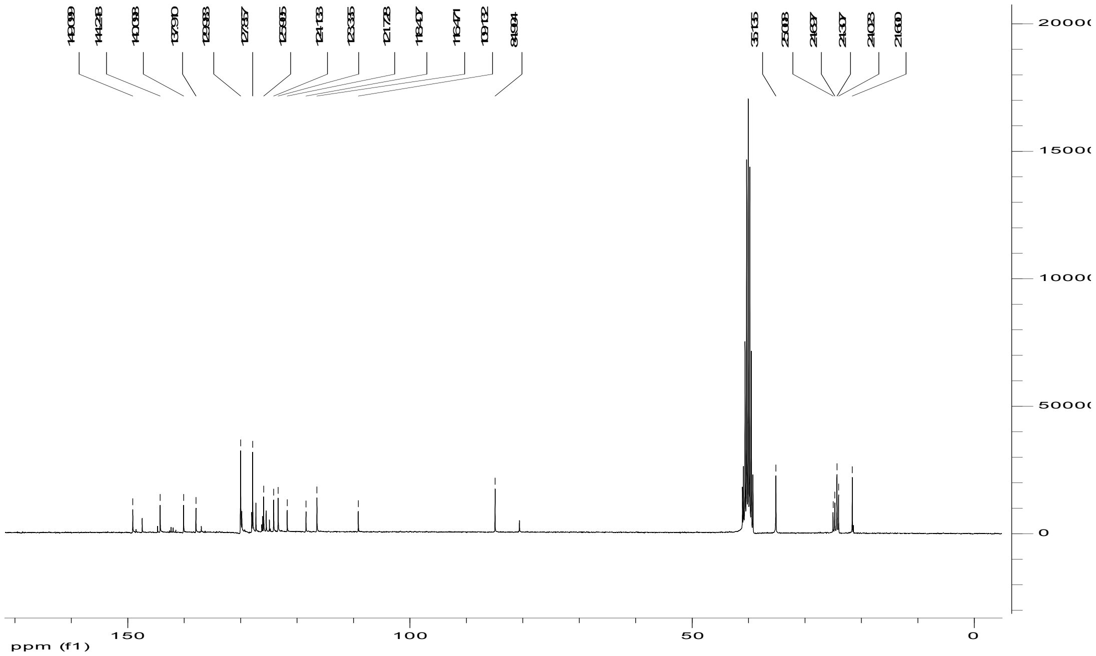

The HRMS was not obtained.
